# Evidence for a liquid silicate layer atop the Martian core

**DOI:** 10.1038/s41586-023-06586-4

**Published:** 2023-10-25

**Authors:** A. Khan, D. Huang, C. Durán, P. A. Sossi, D. Giardini, M. Murakami

**Affiliations:** 1 Institute of Geochemistry and Petrology, ETH Zürich, Zurich, Switzerland; 2 Institute of Geophysics, ETH Zürich, Zurich, Switzerland

**Keywords:** Inner planets, Geophysics, Seismology, Geochemistry

## Abstract

Seismic recordings made during the InSight mission^1^ suggested that Mars’s liquid core would need to be approximately 27% lighter than pure liquid iron^2,3^, implying a considerable complement of light elements. Core compositions based on seismic and bulk geophysical constraints, however, require larger quantities of the volatile elements hydrogen, carbon and sulfur than those that were cosmochemically available in the likely building blocks of Mars^4^. Here we show that multiply diffracted P waves along a stratified core–mantle boundary region of Mars in combination with first-principles computations of the thermoelastic properties of liquid iron-rich alloys^3^ require the presence of a fully molten silicate layer overlying a smaller, denser liquid core. Inverting differential body wave travel time data with particular sensitivity to the core–mantle boundary region suggests a decreased core radius of 1,675 ± 30 km associated with an increased density of 6.65 ± 0.1 g cm^−3^, relative to previous models^2,4–8^, while the thickness and density of the molten silicate layer are 150 ± 15 km and 4.05 ± 0.05 g cm^−3^, respectively. The core properties inferred here reconcile bulk geophysical and cosmochemical requirements, consistent with a core containing 85–91 wt% iron–nickel and 9–15 wt% light elements, chiefly sulfur, carbon, oxygen and hydrogen. The chemical characteristics of a molten silicate layer above the core may be revealed by products of Martian magmatism.

## Abstract (web summary)

Using multiply diffracted P waves and first-principles computations of the thermoelastic properties of liquid iron-rich alloys, we show that the core of Mars is smaller and denser than previously thought.

## Main

Nearly four years of seismic monitoring^9^ appears to suggest that Mars has a large (25% of its total mass) but low-density (6.0–6.3 g cm^−3^) core^2,5–8^. This would imply a significant admixture of light elements of which the most likely, in order of abundance, are S, C, O and H (refs. ^3,4,10–12^). Because these light elements are all cosmochemically volatile, a low-density Martian core suggests that it may have formed before the nebular gas had dispersed^4^, that is, within the first few million years after the condensation of the first solids from the solar nebula, the calcium–aluminium-rich inclusions in chondritic meteorites (taken as *t*_0_ (ref. ^13^)). Indeed, the rapid formation of Mars, within 9 Myr of *t*_0_, is also attested to by Hf–W systematics^14–16^.

However, estimates for the composition of the Martian core have been previously based mainly on either geochemical grounds^11,17,18^ or on seismic and geophysical observations of the mean core density and tidal response of Mars^4–8^. Moreover, the plausible candidates likely to make up the density deficit inferred for the Martian core are all moderately-to-highly volatile, meaning the quantities seen in Martian precursor material are variable and poorly constrained^19,20^. As such, there is lack of knowledge as to the identity and abundance of the predominant light elements in the Martian core.

In view of the recent observations of a P wave diffracted along the core–mantle boundary (CMB)^21,22^ and P waves traversing the core^2^ from two far-side events^21^, the causes of the low mean Martian core density can now be addressed quantitatively from the point of view of both P-wave velocity and density. However, our ability to translate these measurements into composition and temperature is limited by the lack of experimental measurements of the physical properties (P-wave velocity and density) of liquid Fe–Ni–X alloys, where X = S, C, O or H, at conditions relevant to Mars’s core.

To better identify core composition and, in turn, the internal structure of Mars, we combine inversions based on InSight seismic data with ab initio molecular dynamics (AIMD) simulations of the thermoelastic properties of liquid Fe–Ni–X (X = S, C, O or H) mixtures at the pressure–temperature conditions of the Martian core^3^. This is then used to show that the region originally identified as the CMB^2,5–8^ instead more likely represents lowermost molten mantle material that overlies a smaller and denser liquid metallic core. In support of this, we identify seismic phases in the InSight data that are unique products of a molten silicate layer overlying a liquid metallic core.

## Density deficit of the Martian core

**Extended Data Fig. 1:**
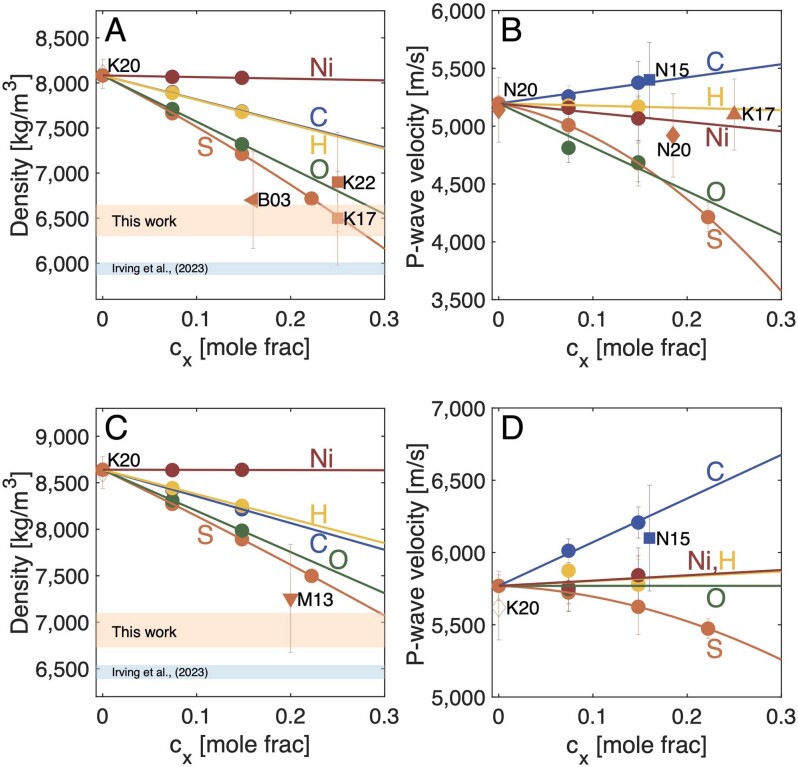
Seismic core properties in the binary system. Density (a,c) and bulk sound velocity (b,d) of liquid Fe-X, where X = Ni, S, O, C and H as a function of alloying element (X) concentration (c_X_) at conditions equivalent of the Martian core-mantle-boundary (19 GPa and 2100 K) and inside Mars’s liquid core (35 GPa and 2400 K) based on the ab inito molecular dynamics simulations (AIMD) of^3^ (solid circles). Experimental data for liquid Fe (K20^73^ and N20^74^), Fe-S (B03^35^, M13^75^, K17^36^, N20^74^ and K22^37^) and Fe-C (N15^26^) are shown using solid and open angular markers. Error bars are reported using 2*σ* for both calculations and experiments, and are visible when exceeding symbol size. For purposes of illustration, only non-spin-polarised AIMD simulations are shown. Similar results are obtained using the spin-polarised AIMD simulations as indicated in Fig. 1 in the main text. Only experiments performed at high pressures (19–35 GPa) are compared through interpolation. Modified from^3^.

Using the equation of state (EoS) derived by ref. ^3^, P-wave velocities and densities of multicomponent Fe–X binary mixtures were computed by varying the mole fractions of Ni, S, C, O and H for a range of pressure and temperature conditions consistent with Mars’s core (Methods and Extended Data Fig. 1).

**Fig. 1:**
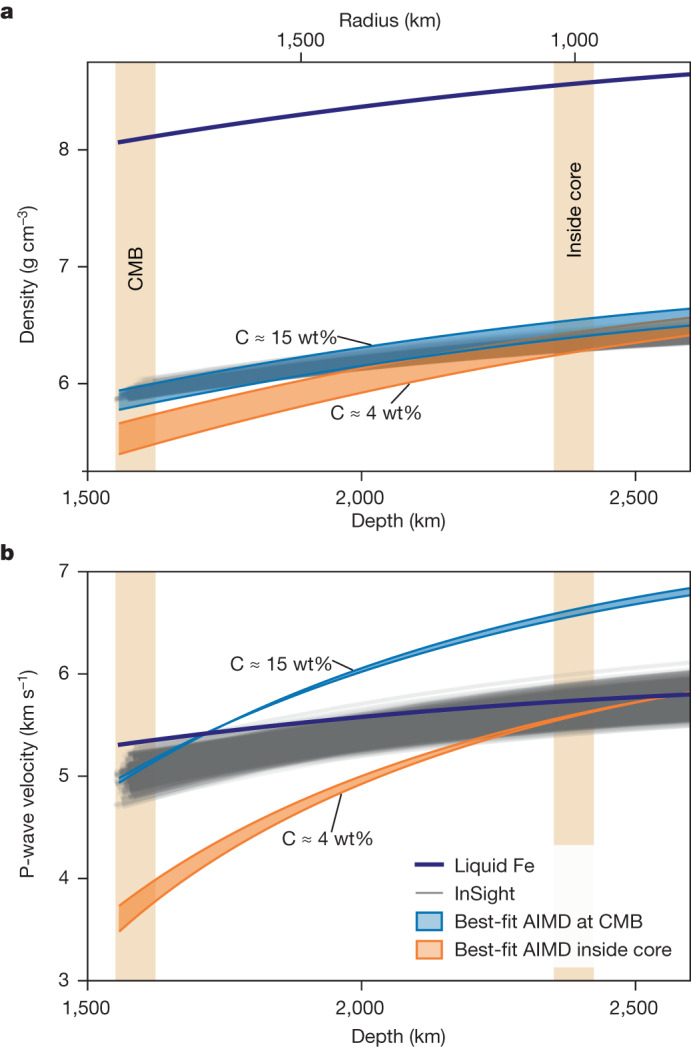
Seismic properties of the core as seen with InSight and from first-principles simulations. Comparison of seismic profiles from InSight with results from first-principles simulations. **a**,**b**, Density (**a**) and P-wave velocity (**b**) profiles of liquid Fe and liquid Fe–Ni–S–C–O–H senary mixtures in Mars’s core obtained from AIMD simulations and InSight observations. The blue shaded profiles represent the predicted seismic core properties based on magnetic (that is, spin-polarized) and non-magnetic (that is, non-spin-polarized) AIMD simulations^3^, including the effects of variations in temperature (±200 K), for the core composition (comprising 67.7% Fe, 5.5% Ni, 7.2% S, 15.1% C, 3.8% O and 0.7% H by weight) that best fits the mean of the InSight seismic profiles within ±2*σ* at the CMB (indicated by the light orange vertical bar labelled ‘CMB’), corresponding to a radius in the range 1,790–1,840 km (ref. ^2^). The orange shaded profiles represent the predicted seismic core properties based on magnetic and non-magnetic AIMD simulations, including the effects of variations in temperature (±200 K), for the core composition (comprising 69.9% Fe, 5.7% Ni, 14.6% S, 4.3% C, 4.7% O and 0.8% H by weight) that best fits the mean of the InSight seismic profiles within ±2*σ* at approximately 800 km in the core (indicated by the light orange vertical bar labelled ‘Inside core’). The labelled C values indicate the C contents of the best-fitting solutions at the CMB and at a depth of around 800 km in the core, respectively. As the orange and blue profiles do not overlap, no single composition exists that matches the InSight observations simultaneously at the CMB and in the core (Methods and Extended Data Fig. 4). Note that the width of the vertical light orange bars has no physical significance.

The first step is to compare the AIMD-predicted P-wave velocity and density profiles for pure liquid Fe with those determined using the InSight seismic data^2^ (Fig. 1). The comparison shows that at Mars’s CMB, that is, the 1,780–1,840 km core radius determined by ref. ^2^, the density of pure liquid Fe is 8.1 g cm^−3^, far denser than Mars’s seismically determined liquid Fe-rich core (approximately 6 g cm^−3^) (Fig. 1a). This implies a density deficit of around 27% relative to liquid Fe, which is almost three times that inferred for Earth’s liquid outer core^23–25^ and suggests significant light-element enrichment in Mars’s core^2,4,5^. By contrast, the P-wave velocity of liquid Fe at Mars’s CMB is similar to that determined from InSight observations (Fig. 1b).

The second step involves computing density and P-wave velocity profiles for a range of compositions by randomly creating senary Fe–Ni–S–C–O–H mixtures. These were matched against the seismic profiles (Methods) within 2*σ* to account for the uncertainty associated with both non-spin- and spin-polarized AIMD simulations^3^ (Fig. 1). We find that there is no single composition capable of simultaneously satisfying the seismic observations at the CMB and at approximately 800 km depth inside the core (orange and blue profiles do not overlap at the two locations simultaneously; see also Extended Data Fig. 4). Although our ‘best-fit’ solution matching InSight’s density and P-wave velocity inside the core contains around 4 wt% C (orange shaded profile in Fig. 1), the same composition underestimates densities by roughly 8% and P-wave velocities by roughly 25% at Mars’s CMB. To account for this mismatch requires around 15 wt% C at the CMB (blue shaded profile in Fig. 1), as C is the sole light element whose addition to Fe metal induces an increase in P-wave velocity required to counteract the effects of the density-driven addition of S and O that both decrease P-wave velocity^3,26^ (Extended Data Fig. 1). However, such elevated C contents are at odds with both its solubility at the eutectic point in the Fe–C binary system (around 3.5 wt% C at 20 GPa)^27,28^ and its abundance in bulk Mars inferred from the depletion trend of volatile lithophile elements (less than or equal to 4 wt% C)^4^. Should P-wave velocity constraints be relaxed so as to consider density alone, unphysical C contents in excess of 10 wt% are required, leaving no plausible composition able to fit InSight observations of the Martian core.

On the basis of this mismatch at the CMB, we propose that the top of what was previously identified as the liquid Fe-rich core^2,5–8^ represents the bottom of the silicate mantle, which is molten, implying an increase in CMB depth and corresponding decrease in core radius. Both the silicate layer and underlying core are required to be fully or almost-fully molten, based on the observation of core-reflected S waves from teleseismic marsquakes^5–7^, the large second-degree tidal Love number^29^, which informs us of the rigidity of the planet, and precise measurements of Mars’s rotation^8^. Geodynamic models^30^ suggest it is plausible that the Martian core is overlain by a molten silicate layer.

In the following, we examine whether a molten silicate layer overlying a smaller liquid core of Mars is compatible with both seismic data and bulk geophysical observations of mean planet mass and moment of inertia.

## Body wave travel time inversion

To determine the seismic properties of a potential molten silicate layer, we invert the updated differential travel time dataset (Methods) presented herein (Supplementary Table 2) and stacked P-to-s waveform (Ps RF) in combination with bulk geophysical observations of mean planet mass and moment of inertia for the radial seismic wave velocity structure and epicentral distance for each event (Extended Data Table 1).

**Fig. 2:**
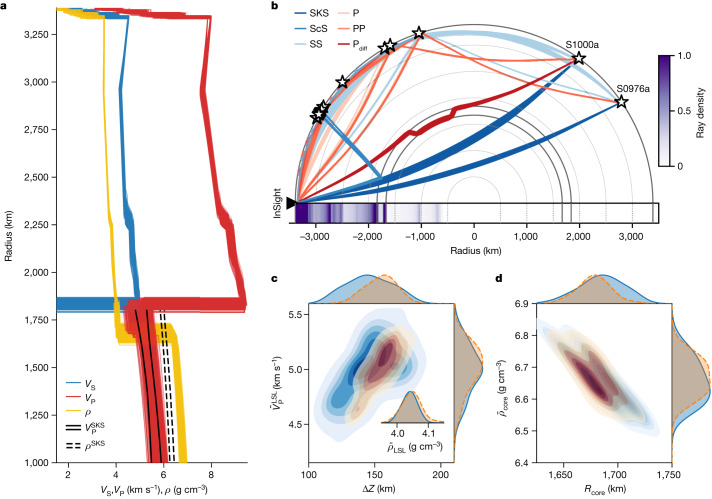
Summary of Mars’s interior structure. **a**, Inverted S- and P-wave velocity and density profiles. For comparison, black solid and dashed lines represent the range of core profiles determined previously using seismic core-transiting (SKS) data^2^. **b**, Body wave ray path geometry for all events (labelled with stars) considered in this study. Colour bar denotes ray path density, that is, the number of rays passing through a given area, based on the inverted models shown in **a**, which explains the diffuseness of the ray paths and source locations. The horizontal column below ‘InSight’ is the radial sensitivity and computed as the integrated ray path density with epicentral distance. Note that the SKS phase for event S0976a is only predicted and not inverted (see Supplementary Information section 1 for details). **c**, Inverted molten silicate layer (LSL) properties (in blue): mean density (${\bar{\rho }}_{{\rm{LSL}}}$), mean P-wave velocity (${\bar{V}}_{{\rm{P}}}^{{\rm{LSL}}}$) and thickness (Δ*Z*). **d**, Inverted core properties (in blue): mean density (${\bar{\rho }}_{{\rm{core}}}$) and core radius (*R*_core_). The probability contours shown in orange in **c** and **d** have been obtained by downsampling the models to additionally match the observed diffracted P-wave reverberation (${{{\rm{P}}}_{{\rm{d}}{\rm{i}}{\rm{f}}{\rm{f}}}}^{\hat{}}{\rm{L}}{\rm{S}}{\rm{L}}\,{{\rm{P}}}_{{\rm{d}}{\rm{i}}{\rm{f}}{\rm{f}}}$) in the LSL (see Fig. 3c and main text for details). Blue- and orange-shaded distributions on top and to the right of **c** and **d** indicate sampled probability distributions for the various parameters shown in the plots.

The results from the inversion are plotted in Fig. 2 (thermal models are shown in Extended Data Fig. 5). Differential travel time, Ps RF and bulk geophysical data fits are shown in Extended Data Fig. 6. In contrast to published geophysical models (for example, ref. ^5^), we find that the core-diffracted P wave arrival can be fit if a fraction of Mars’s CMB region, previously believed to be the core^2,5–7^, is allocated to a liquid silicate layer (LSL) with P_diff_ diffracting along its lower rather than its upper boundary (Fig. 2b) as considered previously^22^. The radial seismic core profiles (Fig. 2a) are compared to the models from ref. ^2^ and indicate good agreement in terms of core P-wave velocity structure, but, as expected, differences in density since the outermost core now represents the lowermost mantle.

The LSL is characterized by P-wave speeds and densities ranging between 4.5–5.5 km s^−1^ and 4.0–4.1 g cm^−3^ (Fig. 2c), respectively, which only slightly exceed our AIMD predictions for liquid silicates (Extended Data Table 3) based on a primitive Martian shergottite^31^ and the mantle composition of ref. ^4^ for which P-wave speeds and densities range from 4.8–5.2 km s^−1^ and 3.7–3.8 g cm^−3^, respectively. The thickness of the layer determined by the geophysical inversions performed here is 145 ± 25 km and is, as expected, correlated with mean LSL P-wave speed. Thinner, higher-density and higher-P-wave-speed layers or thicker, lower-density and lower-P-wave-speed layers beyond the mapped ranges of LSL properties obtained here are incompatible with the bulk geophysical observations and seismic data.

The LSL density derived here (approximately 4 g cm^−3^) precludes a stably stratified Fe–Ni–light-element-enriched layer at the top of the core as suggested for the Earth (for example, ref. ^32^). In turn, the presence of the LSL implies that the radius of the liquid metallic core is smaller (1,640–1,740 km) (Fig. 2d) than recently reported (1,780–1,840 km)^2^. Consequently, mean core density increases to 6.65 ± 0.15 g cm^−3^, which is denser than earlier estimates that ranged between 6.0–6.3 g cm^−3^ (refs. ^2,4,6^).

While the LSL thus has profound implications for the nature of the core and bulk composition of Mars, the radial seismic models (Fig. 2a) obtained from inversion of differential travel times, mass and moment of inertia are, however, non-unique^22^ and therefore insufficient as a means of unequivocally establishing the presence of the LSL. Consequently, we query the InSight seismic data for evidence that allows for an independent confirmation of the existence of the LSL.

## Seismic evidence for a molten layer

**Fig. 3:**
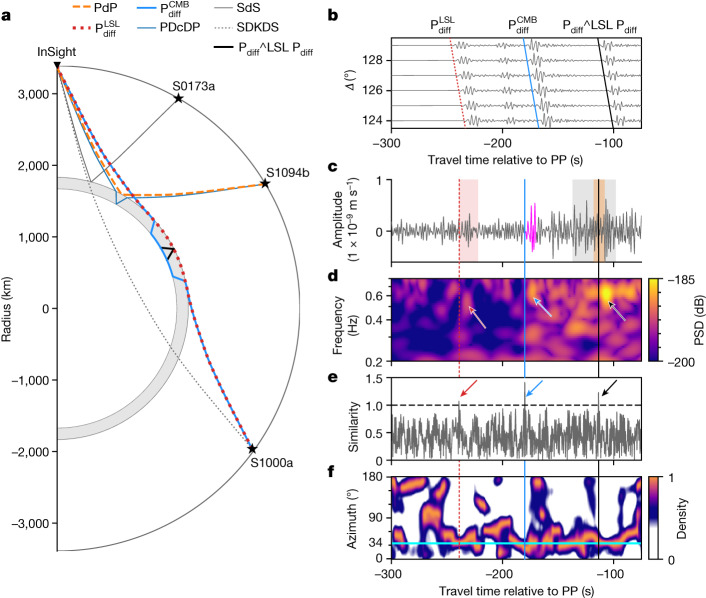
Molten silicate layer  and core seismic signatures. **a**, Ray paths for LSL- and core-interacting phases: P wave and S wave reflected from the top of the LSL (grey layer) (PdP and SdS), P wave diffracted around the mantle–LSL interface (${{\rm{P}}}_{{\rm{diff}}}^{{\rm{LSL}}}$), P wave reflected from the liquid core (PDcDP), P wave diffracted around the LSL–liquid core interface (${{\rm{P}}}_{{\rm{diff}}}^{{\rm{CMB}}}$) and reverberating in the LSL (${{{\rm{P}}}_{{\rm{d}}{\rm{i}}{\rm{f}}{\rm{f}}}}^{\hat{}}{\rm{L}}{\rm{S}}{\rm{L}}\,{{\rm{P}}}_{{\rm{d}}{\rm{i}}{\rm{f}}{\rm{f}}}$), and liquid-layer and core-transiting P wave (SDKDS). **b**, Vertical-component synthetic waveform section showing the diffracted P wavetrain for epicentral distances similar to S1000a (126°, see Supplementary Fig. 7 for a larger section). **c**, Vertical-component observed polarized waveforms (filtered between 0.2–0.7 Hz) and envelopes showing the P_diff_ arrivals, marked by red, blue and black lines, respectively. The vertical-component template trace employed for waveform matching (**e**) is shown in magenta and consists of a 10-s-long window including the observed ${{\rm{P}}}_{{\rm{diff}}}^{{\rm{CMB}}}$ arrival. Red- and grey-shaded rectangles represent the travel time predictions for ${{\rm{P}}}_{{\rm{diff}}}^{{\rm{LSL}}}$ and ${{{\rm{P}}}_{{\rm{d}}{\rm{i}}{\rm{f}}{\rm{f}}}}^{\hat{}}{\rm{L}}{\rm{S}}{\rm{L}}\,{{\rm{P}}}_{{\rm{d}}{\rm{i}}{\rm{f}}{\rm{f}}}$, respectively, based on the inverted models shown in Fig. 2, and the yellow-shaded rectangle spans the range satisfying the observed differential travel time (−113 ± 5 s) of ${{{\rm{P}}}_{{\rm{d}}{\rm{i}}{\rm{f}}{\rm{f}}}}^{\hat{}}{\rm{L}}{\rm{S}}{\rm{L}}\,{{\rm{P}}}_{{\rm{d}}{\rm{i}}{\rm{f}}{\rm{f}}}$ relative to PP. **d**, Three-component scalogram illustrating the temporal change in frequency content. ${{\rm{P}}}_{{\rm{diff}}}^{{\rm{LSL}}}$, ${{\rm{P}}}_{{\rm{diff}}}^{{\rm{CMB}}}$ and ${{{\rm{P}}}_{{\rm{d}}{\rm{i}}{\rm{f}}{\rm{f}}}}^{\hat{}}{\rm{L}}{\rm{S}}{\rm{L}}{{\rm{P}}}_{{\rm{d}}{\rm{i}}{\rm{f}}{\rm{f}}}$ arrivals are indicated by arrows following the colour scheme in **c**. **e**, Similarity between event trace and template trace. The horizontal line designates the threshold employed for the waveform matching detections. Coloured arrows as in **d**. **f**, Polarization attributes for the three-component seismic data, showing the temporal change in azimuth between 0.2–0.7 Hz. The azimuth across the observed diffracted P wavetrain is consistent with the imaged meteorite impact location of 34° (horizontal cyan line)^33^. Supporting seismic waveform processing information is provided in Supplementary Information section 6. S0173a, S1000a and S1094b: locations of a marsquake and two imaged meteorite impacts (Extended Data Table 1).

Potential seismic phases interacting with the LSL and the core are shown in Fig. 3. Relative to Fig. 2b, we have modified the nomenclature of the seismic phases to distinguish those that reflect off of, traverse or otherwise interact with the LSL. To search for these phases, we consider a near- (S1094b) and a far-side (S1000a) imaged impact event^33^. We first performed synthetic waveform analyses of models with and without LSLs (Supplementary Figs. 7 and 8) to qualitatively assess their seismic predictions. Most notably, several LSL-interacting phases are found that are not present in the published model without the LSL.

The S wave reflected from the top of the LSL (SdS) has been identified in waveforms from events that cluster in and around Cerberus Fossae (here illustrated by event S0173a), but represents an S-wave reflection from the top of the LSL, rather than a core reflection (ScS) as reported earlier^5–7^, and anchors the location of the top boundary of the LSL. SDKDS corresponds to a mantle-traversing S wave that converts at and transits the LSL (D) and liquid core (K) as a P wave (previously denoted SKS in ref. ^2^ and Fig.  2b). Both SdS-P and SDKDS-PP differential travel time measurements are fit within uncertainties with our new model (Extended Data Fig. 6).

As a consequence of the impedance contrasts in P-wave speed and density at the top and bottom of the LSL, respectively, synthetic diffracted P waves on both mantle–LSL (${{\rm{P}}}_{{\rm{diff}}}^{{\rm{LSL}}}$) and LSL–liquid core (${{\rm{P}}}_{{\rm{diff}}}^{{\rm{CMB}}}$; denoted P_diff_ in Fig. 2b after ref. ^22^) interfaces exist, in addition to a reverberation within the layer (${{{\rm{P}}}_{{\rm{d}}{\rm{i}}{\rm{f}}{\rm{f}}}}^{\hat{}}{\rm{L}}{\rm{S}}{\rm{L}}\,{{\rm{P}}}_{{\rm{d}}{\rm{i}}{\rm{f}}{\rm{f}}}$), all with similar move-out, waveform and differential travel time relative to ${{\rm{P}}}_{{\rm{diff}}}^{{\rm{CMB}}}$ (Fig. 3b and Supplementary Fig. 7). In line with this, inspection of the observed waveform from the impact event S1000a (Fig. 3c) and scalogram (Fig. 3d) shows, in addition to ${{\rm{P}}}_{{\rm{diff}}}^{{\rm{CMB}}}$, two arrivals with waveforms that match ${{\rm{P}}}_{{\rm{diff}}}^{{\rm{CMB}}}$ and similar differential travel time relative to ${{\rm{P}}}_{{\rm{diff}}}^{{\rm{CMB}}}$ (Fig. 3e), and near-identical polarization (Fig. 3f). Moreover, the polarization of the observed P_diff_ wavetrain is largely consistent with the imaged location of the impact^33^ (indicated by the cyan line in Fig. 3f). The diffracted body wave phases reported here are verified independently using narrow-band-filtered time-domain polarized waveforms and envelopes (Supplementary Information section 6). Another synthetically predicted phase with similar waveform but different move-out is seen to arrive between ${{\rm{P}}}_{{\rm{diff}}}^{{\rm{LSL}}}$ and ${{\rm{P}}}_{{\rm{diff}}}^{{\rm{CMB}}}$ (Fig. 3b), but is too weak to be identified in the observed data. Diffracted S waves also could not be identified (Supplementary Information section 6.3).

The observation of LSL-interacting seismic phases allows for improved determination of LSL thickness by downsampling the inverted models (Fig. 2) to fit ${{{\rm{P}}}_{{\rm{d}}{\rm{i}}{\rm{f}}{\rm{f}}}}^{\hat{}}{\rm{L}}{\rm{S}}{\rm{L}}\,{{\rm{P}}}_{{\rm{d}}{\rm{i}}{\rm{f}}{\rm{f}}}$ (${{\rm{P}}}_{{\rm{diff}}}^{{\rm{LSL}}}$ is insensitive to LSL properties) within the observational uncertainties (yellow-shaded rectangle in Fig. 3c). As a consequence, the estimate of LSL thickness improves to 150 ± 15 km (1*σ*, red contours in Fig. 2c), while core density and radius are slightly modified to 6.65 ± 0.1 g cm^−3^ and 1675 ± 30 km (1*σ*, red contours in Fig. 2d), respectively.

Finally, P waves reflected from the top (PdP) and bottom (PDcDP) of the LSL are small-amplitude P-wave phases that arrive in the PP-wave coda (Supplementary Fig. 7) and are challenging to identify in the observed waveforms (Supplementary Fig. 9d,e) from event S1094b. We looked at waveforms from other low-frequency events that cluster around the seismically most-active region (Cerberus Fossae) discovered thus far^34^, but, because of the interference of S-wave coda (Supplementary Fig. 11), we are unable to positively identify PdP and PDcDP phases.

## Core composition

**Fig. 4:**
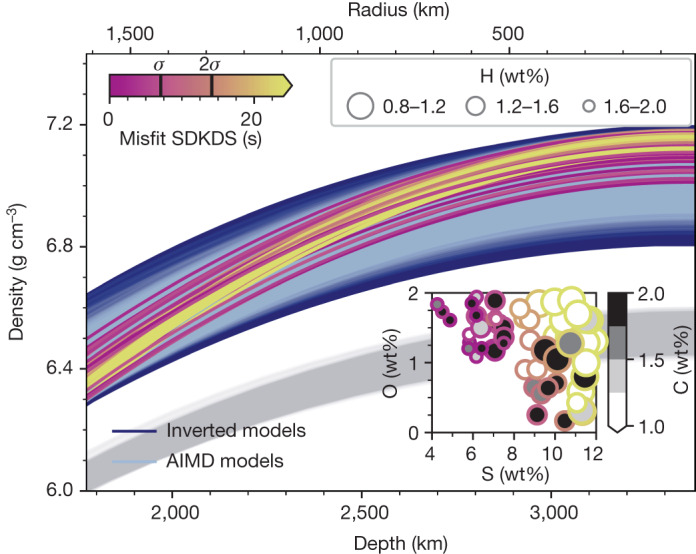
Mars’s core composition and light-element budget. AIMD-predicted core density profiles (AIMD models^3^) for senary Fe–Ni–S–C–O–H mixtures (light blue lines) that match the inverted seismic core density profiles obtained here (dark blue lines). For comparison, the InSight density profiles from ref. ^2^ based on the larger core radius of 1,780–1,840 km are also shown (light grey lines). The inset shows the senary Fe–Ni–S–C–O–H core compositions after additional application of cosmochemical constraints (see main text for details). For comparison, the entire range of core compositional models before application of the cosmochemical constraints (corresponding to all the light blue AIMD-predicted density profiles) is shown in Supplementary Fig. 13. Each core composition in the inset is further labelled (coloured circle) by its residual (misfit) between observed and AIMD-computed differential core- and LSL-transiting (SDKDS) travel time (ray path is shown in Fig. 3a). The corresponding density profiles are colour-coded accordingly.

In light of the evidence for a smaller and denser Martian core, we return to the question of its composition. To constrain the light-element budget of the core, we randomly created 10^8^ senary Fe–Ni–S–C–O–H mixtures using our mixing model (Methods), computed density profiles for each, and selected those that matched our seismically determined density models. The resultant set of AIMD-based density models are shown in Fig. 4. Relying exclusively on density represents a more robust approach that is driven by the agreement in the calculation of density between AIMD simulations and experimental observations^3^. As a means of accounting for the uncertainties associated with experimental density measurements (Extended Data Fig. 1), we varied the EoS for the density of Fe–S within the experimental errors of refs. ^35–37^ in our AIMD simulations.

Cosmochemical arguments (Methods) place additional constraints on the Martian core composition and result in plausible total light-element abundances in the range 9–15 wt% (see inset in Fig. 4). The models are further colour-coded according to their ability to match the observed SDKDS travel time (Fig. 3a). Yet, owing to the uncertainty in AIMD- and experimentally derived P-wave velocity^3^, we use the SDKDS residual only as an indicator, rather than a filter to select compositions. Although models with reasonable amounts of S, C and O abound, H abundances exceed 1 wt%, higher than in mixtures of the plausible chondritic precursors of Mars, which contain at most 0.3 wt% H (ref. ^4^). This implies that the partition coefficient between liquid metal and silicate during core formation is at least 3, which is within the range of theoretical predictions^38^ but higher than experimental determinations^39^. Should the partition coefficient fall in the lower end of this range then the high apparent H contents found here imply the presence of additional elements (for example, N and P), which would lower the need for H in Mars’s core.

## Implications for interior structure

Our new seismic model resolves the issue of the low mean core density of Mars that implied a light-element complement in excess of 20 wt% (refs. ^2,3,5–7^), which is too high relative to their cosmochemical availability in the potential building blocks that accreted to form Mars^4,12^. An important caveat is that we assume the LSL to be global and interconnected, based on having imaged parts of the lowermost mantle and CMB region. A local LSL is, in any case, expected to be dynamically unstable in the sense that it would either spread globally to cover the core or become entrained with upwelling mantle material. While topographic variations on the mantle–LSL interface are not considered on account of the paucity of lower-mantle-sensitive seismic P-wave phases, a locally thinner LSL cannot entirely be excluded in view of the fact that our predictions for ${{\rm{P}}}_{{\rm{diff}}}^{{\rm{LSL}}}$ arrive marginally late. Yet, any variations in topography on the mantle–LSL interface must be weak as the diffracted P-wave arrivals would otherwise be obscured, contradicting our observations. However, the fact that ${{\rm{P}}}_{{\rm{diff}}}^{{\rm{LSL}}}$ is also affected by lower mantle P-wave velocity structure convolutes the problem of disentangling the various contributions on the basis of the present observations.

The observation that no single composition is capable of simultaneously fitting current seismic properties in the shallow and deep parts of the core (Fig. 1) ultimately led to our revision of its density and radius. Yet, we should emphasize that our new interior structure model of Mars is derived on the basis of seismic observations, independently of any AIMD-computed properties. While the AIMD simulations bridge a gap in our current understanding of the physical properties of liquid Fe–Ni–X alloys, they must eventually be verified by experiments. The AIMD simulations further indicate that the densities of liquid primitive shergottite or bulk silicate Mars compositions (Extended Data Table 2), including solid mantle, are lower than those of the LSL. The roughly 0.2 g cm^−^^3^ excess density found here relative to our AIMD predictions for the aforementioned silicate compositions probably results from FeO enrichment in the LSL relative to the bulk Martian mantle.

For a molten layer to remain dynamically stable, density and viscosity contrasts of greater than or equal to 80 kg m^−^^3^ and approximately 100, respectively, between liquid and solid mantle, are required^30,40,41^, in agreement with our results. The temperature at which the liquid layer remains molten as a function of its Mg number (= Mg/Mg + Fe^2+^) dictates its time-integrated stability. For temperatures around 2,000–2,300 K obtained in our inversions (Extended Data Fig. 5), this corresponds to Mg numbers of 0.2–0.3 (Supplementary Information section 9). We speculate that low Mg numbers may reflect chemical exchange with the underlying liquid core, or represent a vestige of magma ocean crystallization on Mars, which, under reducing conditions, would have resulted in increasing FeO in the liquid as solidification proceeded^42^. Moreover, in this scenario, the layer would also be enriched in heat-producing elements (U, Th and K) that are highly incompatible during crystalliszation, providing an additional heat source to maintain a molten layer^30^. We therefore propose that the chemical and isotope signature of such a layer may have been preserved in melting products of the Martian analogues of mantle plumes that would have risen from the thermal boundary layer surrounding the core and entrained fractions of the LSL (for example, refs. ^43–45^). Variations in Hf–W and highly siderophile element systematics may be detectable in plume-derived partial melts .

## Methods

### Mixing of multicomponent liquid Fe–Ni–X alloys

Ideal mixing of end-member liquid Fe–Ni–X (X = S, C, O or H) alloys has been widely practised (for example, refs. ^24,46,47^) to evaluate the elastic properties of multicomponent Fe-rich liquids. Ideal mixing has been confirmed under Earth’s core conditions^48^; however, at lower pressures, our simulations^3^ show non-ideal behaviour of density and incompressibility when mixing Fe and S (Extended Data Fig. 1). As a first step, we design a mixing model by accounting for the non-ideality of the Fe–S binary.

Density (*ρ*) and isothermal bulk modulus (*K*_*T*_) of the Fe–Ni–X mixture were computed at two *P*–*T* conditions based on the model of ref. ^3^: at 19 GPa and 2,100 K (equivalent of the CMB as determined by ref. ^2^) and at 35 GPa and 2,400 K (corresponding to around 2,800 km depth) using the expressions:

1 $${\rho }_{{\rm{m}}{\rm{i}}{\rm{x}}}({x}_{i})={\rho }_{{\rm{F}}{\rm{e}}}+\mathop{\sum }\limits_{i}^{n}{\int }_{0}^{{x}_{i}}\frac{{\rm{\partial }}\rho }{{\rm{\partial }}{x}_{i}}{\rm{d}}{x}_{i},$$

and

2 $${K}_{{T}_{{\rm{m}}{\rm{i}}{\rm{x}}}}({x}_{i})={K}_{{T}_{{\rm{F}}{\rm{e}}}}+\mathop{\sum }\limits_{i}^{n}{\int }_{0}^{{x}_{i}}\frac{{\rm{\partial }}{K}_{T}}{{\rm{\partial }}{x}_{i}}{\rm{d}}{x}_{i},$$

where *x*_*i*_ is the concentration (in molar fraction) of the impurity elements (*n* = 5, viz. Ni, S, O, C and H) and *ρ*_Fe_ and ${K}_{{T}_{{\rm{Fe}}}}$ are the density and isothermal bulk modulus of pure liquid Fe, respectively. As indicated in Extended Data Fig. 1, partial derivatives of *ρ* and *K*_*T*_ with respect to *x*_*i*_ are constant within the modelling uncertainties of the simulations for Ni, O, C and H, except for S because of its nonlinear behaviour^3^. The P-wave velocity (*V*_P_) of the mixture is obtained from:

3 $${V}_{{\rm{P}}}=\sqrt{\frac{{K}_{S}}{\rho }},$$

with

4 $${K}_{S}=(1+\alpha \gamma T){K}_{T},$$

where *K*_*S*_ is the adiabatic bulk modulus, *α* is the volumetric coefficient of thermal expansion, *γ* is the thermodynamic Grüneisen parameter, *T* is temperature and *K*_*T*_ and *ρ* are density and isothermal bulk modulus of the liquid mixtures, respectively. Values for *α*, *γ* and *T* for our mixing model are taken from ref. ^3^. The fact that we assume *α* and *γ* to be constant with *P* and *T*, that is, independent of species and concentration of the impurity element *x*_*i*_, might serve as a source of uncertainty in the mixing model, but is nevertheless supported by the negligible change (within uncertainties) of *α* and *γ* upon introduction of light elements into liquid Fe^49^.

### Matching density and P-wave velocity in the core

Relying on the AIMD simulations of ref. ^3^, the mixing model (equations (1)–(3)) allows one to fully map the compositional space onto the density and velocity spaces at the two *P*–*T* conditions at which our simulations are anchored. By reproducing the observed density and P-wave velocity at these two locations, we are able to propose a range of compositions that are consistent with the InSight observations within 2*σ* to account for the uncertainty associated with both non-spin- and spin-polarized AIMD simulations. In the following, we evaluate the feasibility of these compositions in the light of geo- and cosmochemical considerations.

#### Mars’s core contains a single light element: Fe–Ni–X

To demonstrate the procedure, we begin with the simplest scenario, which is the Fe–Ni–X ternary system, that is, the liquid core is composed of Fe–Ni and a single light element (S, O, C or H). Following the same cosmochemical and geophysical arguments as ref. ^4^, and because of its negligible influence on the elasticity of Fe, all our mixing models keep the Fe/Ni mass ratio in Mars’s core constant at 12.36. While instructive, the ternary case is readily refuted by the 27% density deficit and similar P-wave velocity at Mars’s CMB, relative to pure liquid Fe (ref. ^3^). For instance, and as indicated in Extended Data Fig. 1, the most efficient element in reducing the density of liquid Fe is S, which, however, would have to comprise at least 30 mol% of the mixture, leading to a mismatch with P-wave velocity; the same applies to O, which affects P-wave velocity and density similarly. C can also be excluded as a single light element because it increases P-wave velocity, whereas the effect of H on P-wave velocity is negligible.

#### Mars’s core contains two light elements: Fe–Ni–S–X

**Extended Data Fig. 2:**
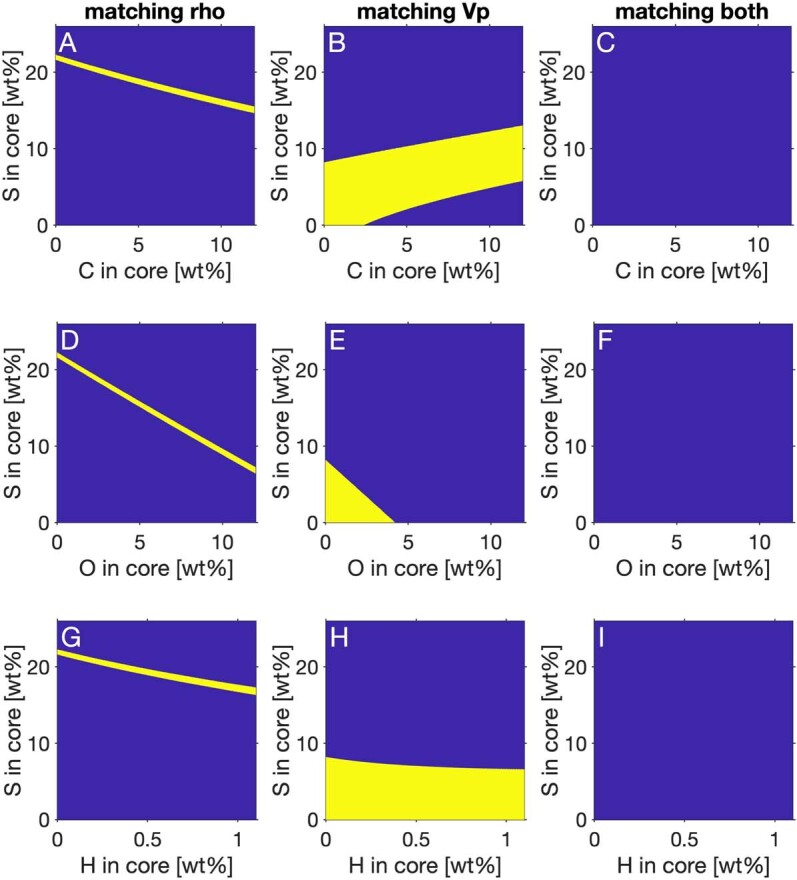
Matching core seismic properties in the quaternary system at the core-mantle-boundary. Range of core compositions in the liquid Fe-Ni-S-X (X = C, O, H) quaternary system (blue regions) that match InSight observations (yellow regions) of core density (left column, panels a, d, g), core P-wave velocity (middle column, panels b, e, h), and both properties simultaneously (right column, panels c, f, i) at a location just below the core-mantle-boundary. For purposes of illustration, only non-spin polarised ab initio molecular dynamics (AIMD) simulations are shown. Similar results are obtained using the spin-polarised AIMD simulations as indicated in Fig. 1 in the main text.

**Extended Data Fig. 3:**
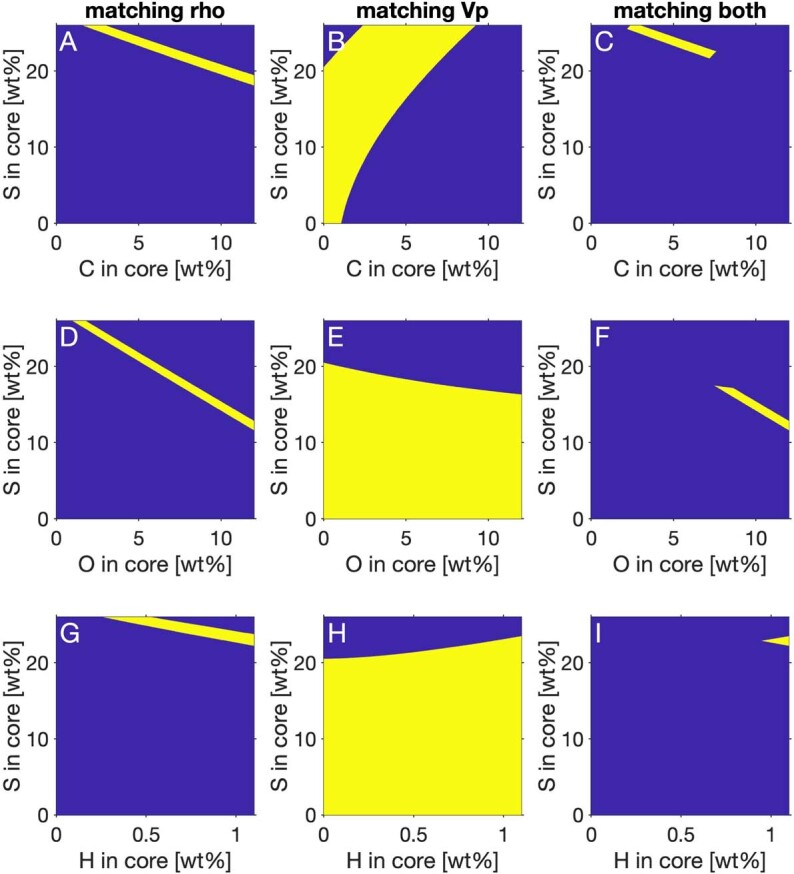
Matching core seismic properties in the quaternary system inside the core. Range of core compositions in the liquid Fe-Ni-S-X (X = C, O, H) quaternary system (blue regions) that match InSight observations (yellow regions) of core density (left column, panels a, d, g), core P-wave velocity (middle column, panels b, e, h), and both properties simultaneously (right column, panels c, f, i) inside the core. For purposes of illustration, only non-spin polarised ab initio molecular dynamics (AIMD) simulations are shown. Similar results are obtained using the spin-polarised AIMD simulations as indicated in Fig. 1 in the main text.

Next, we consider the Fe–Ni–S–X quaternary system, that is, a core containing Fe–Ni–S and another light element (C, O and H). S is widely considered a major light alloying element in Mars’s core^4,18,50–52^. As demonstrated in Extended Data Figs. 2 and 3, it is reasonably easy to find a quaternary Fe–Ni rich mixture that matches either density or P-wave velocity within Mars’s core; yet, it is more difficult to match both properties and is impossible at the two considered *P*–*T* conditions. The quaternary system serves to illustrate first-order relations, as determined by seismic constraints, between S and other light-element candidates, such as the negative S–O correlation.

#### Sampling the entire compositional space: Fe–Ni–S–C–O–H

**Extended Data Fig. 4:**
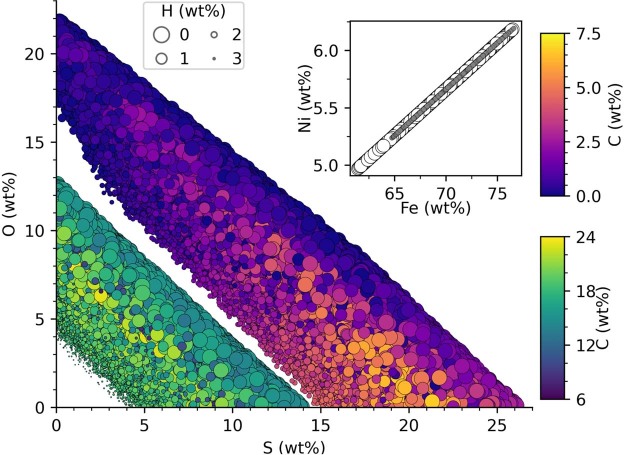
Matching core seismic properties in the senary system. Distribution of liquid Fe-Ni-S-O-C–H senary mixtures that match both density and P-wave velocity in the core immediately below the core-mantle-boundary (CMB) (blue-green-yellow colour bar) and inside the core (blue-purple-orange colour bar), comprising 1.28 ⋅ 10^5^ (1.3‰) and 3.09 ⋅ 10^5^ out of 100 million randomly generated compositions (3.1‰), respectively. The inset shows the distribution of Fe versus Ni at the CMB (white) and inside the core (grey). For purposes of illustration, only non-spin polarised ab initio molecular dynamics (AIMD) simulations are shown. Similar results are obtained using the spin-polarised AIMD simulations as indicated in Fig. 1 in the main text.

**Extended Data Fig. 5:**
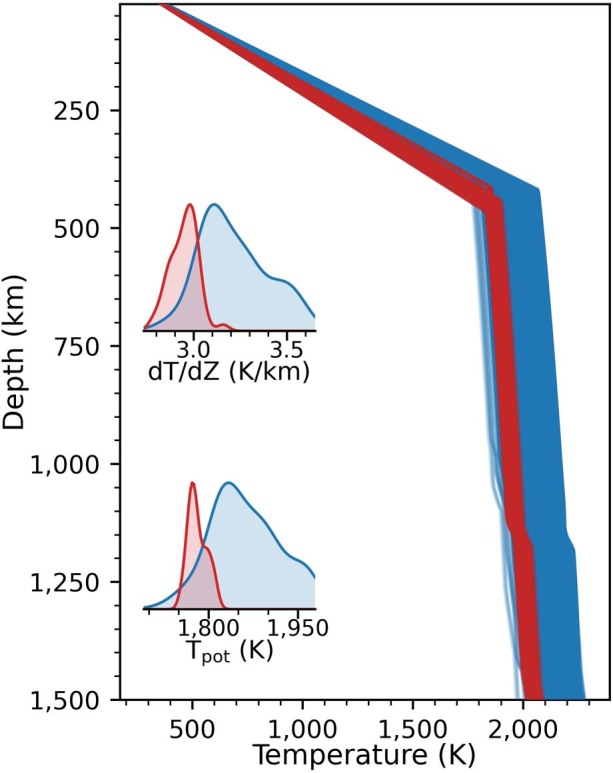
Martian mantle geotherms. Inverted lithospheric and mantle geothermal profiles for high^55–58^ (blue), and low^4^ (red) FeO mantle compositions. The insets show the distributions of sampled potential temperature (T_pot_) and lithospheric geothermal gradient (dT/dz) for high (light blue) and low (light red) FeO compositions.

Relying on our mixing equations (equations (1)–(3)), we randomly generate 10^8^ Fe–Ni–S–O–C–H senary mixtures and compute their density and P-wave velocity at the two prescribed *P*–*T* conditions. By matching the so-computed densities and P-wave velocities to the observed values within 2*σ*, only 1.3‰ and 3.1‰ of the 10^8^ compositions are retained (Extended Data Fig. 4).

The fixed Fe/Ni ratio in Mars’s core defines the trend seen in the inset in Extended Data Fig. 4. Because S and O have similar effects on density and P-wave velocity (Extended Data Fig. 1), particularly at the CMB, their roles in reproducing the observations are almost interchangeable, which leads to a strong negative correlation between S and O as illustrated in Extended Data Fig. 4. While interdependence of S and O in liquid Fe during metal–silicate (core–mantle) equilibration remains debated^53,54^, our observation of S–O dependence provides an independent constraint from a combined geophysical and mineral-physics perspective.

Compared with previous core composition models that were mainly based on cosmochemical data because of lack of seismic measurements^18,55–58^, our core compositions rely fully on InSight seismic data, in addition to geophysical observations that sense the large-scale structure of Mars. Extended Data Fig. 4 shows that there is no unique composition capable of simultaneously satisfying the seismic observations at both *P*–*T* conditions.

The key constraint for the composition of the Martian core is the trade-off between S and C contents required to fit the observed P-wave velocity (Fig. 1 and Extended Data Fig. 4)^3^. Too much S results in velocities that are too slow relative to observations, which is counterbalanced by a complementary fraction of C that acts to increase P-wave velocity relative to liquid Fe metal. The indeterminacy between C and H, on the other hand, reflects the fact that these elements overall affect core properties similarly.

### Thermoelastic properties of Mars’s core

Next, we interpolate and extrapolate *ρ* and *K*_*T*_, and thus the bulk sound velocity *V*_Φ_, given a composition *x*, over the entire Martian core along the aerotherm of ref. ^4^. The effects of pressure and composition are described by equation (1) and a second-order Birch–Murnaghan EoS:

5 $$P=\frac{3}{2}{K}_{0}\left[{\left(\frac{{V}_{0}}{V}\right)}^{\frac{7}{3}}-{\left(\frac{{V}_{0}}{V}\right)}^{\frac{5}{3}}\right]\left[1+\frac{3}{4}\left({K}_{0}^{{\prime} }-4\right)\left({\left(\frac{{V}_{0}}{V}\right)}^{\frac{2}{3}}-1\right)\right],$$

where *K*_0_ is the isothermal bulk modulus, *V*_0_ the reference volume at the CMB reference pressure (19 GPa) and ${K}_{0}^{{\prime} }$ is the first derivative of the bulk modulus with respect to pressure, which equals 4. If not specified otherwise, subscript 0 always refers to CMB pressure. The thermal effect is taken into account through the relationship:

6 $${\rm{d}}\rho (P,x)=-\,\rho (P,x)\alpha (P){\rm{d}}T,$$

where a linear behaviour of *α* with respect to pressure (*P*) is assumed^3^. The influence of temperature and composition on the isothermal bulk modulus are accounted for through ${({\rm{d}}{K}_{T}/{\rm{d}}T)}_{P=0}$ = −0.026 GPa K^−1^ (Extended Data Table 3) and equation (2), while that of pressure is given by:

7 $${K}_{T}(T\,,x)={K}_{T,0}(T\,,x)+{\int }_{0}^{P}\frac{\partial {K}_{T}}{\partial P}{\rm{d}}P\,,$$

where *K*_*T*,0_ is the isothermal bulk modulus at the reference CMB pressure (19 GPa). From equation (5), we have ∂*K*_*T*_/∂*P* = 4, while the isentropic bulk modulus *K*_*S*_ and the bulk sound velocity *V*_Φ_ of the mixture are obtained using equations (3) and (4). As above, we assume *α* to be independent of composition and *γ* to be constant (= 2.7), that is, independent of *P*, *T* and *x*, since our simulations demonstrated the invariability of *γ*, within errors, across the *P*–*T* conditions of Mars’s core (Extended Data Table 3).

### Body wave travel time inversion

To determine the seismic properties of a potential molten silicate layer, we rely on the converted, reflected and diffracted seismic body wave phases P, pP, PP, PPP, P-to-s, P_diff_, S, sS, SS, SSS and ScS described in refs. ^6,22^ and the core-traversing phase (SKS) initially reported in ref. ^2^ and updated here (Supplementary Information section 1) from 15 low-frequency marsquakes and two meteorite impacts^33^ with moment magnitudes *M*_W_ in the ranges 3.0–4.0 and 4.0–4.1 (ref. ^59^), respectively, that cover an epicentral distance (*Δ*) range from approximately 30°–145° (refs. ^6,22,33^). The updated differential travel time dataset (all phases are relative to P or PP) presented herein (Supplementary Table 2) comprise 82 seismic phase picks with bottoming depths to around 2,700 km that we simultaneously invert in combination with bulk geophysical observations of mean planet mass and moment of inertia for the radial seismic wave velocity structure and location (*Δ*) for each event (Extended Data Table 1).

We consider a spherically symmetric model of Mars and assume compositional homogeneity. Mantle seismic properties are computed using a geophysical parameterization that relies on a unified description of phase equilibria, seismic properties and thermochemical parameters^5,6,60^. The seismic properties for the molten silicate layer and core, both of which are assumed to be homogeneous and well-mixed liquids, are obtained using an EoS approach^2^ (details for computing seismic profiles are given below).

Underpinning our work is the assumption that lateral variations in the seismic structure of the Martian interior are negligible, owing to the difficulty of resolving lateral heterogeneities from scarce single-station data. Trials using thermochemical models of Mars’s interior^61^ suggest travel time differences that are commensurate with the observational uncertainty^2^.

### Computing seismic profiles

To set up and run the parameterization, we assume a homogeneous bulk composition for the mantle, considering a number of different model Martian compositions with high and low FeO contents^4,55–58^ (similar results are obtained for models with both high and low FeO contents; compare Fig. 2 and Supplementary Fig. 6), and computed P- and S-wave velocities and density as a function of temperature, composition and pressure using Gibbs free-energy minimization^62^. We further parameterized the Martian geotherm using variable conductive crustal and lithospheric geotherms, whereas the underlying mantle is assumed to be adiabatic. As we rely on a seismic parameterization of the crust, the nature of the crustal geotherm is less significant. Mantle adiabats (isentropes) are computed self-consistently from the entropy of the lithology at the pressure and temperature of the bottom of the thermal lithosphere. Uncertainties in density and elastic moduli computed using Gibbs free-energy minimization are less than 1% and less than 2–4%, respectively^63^. To compute pressure, the load is integrated from the surface assuming hydrostatic equilibrium.

**Extended Data Fig. 6:**
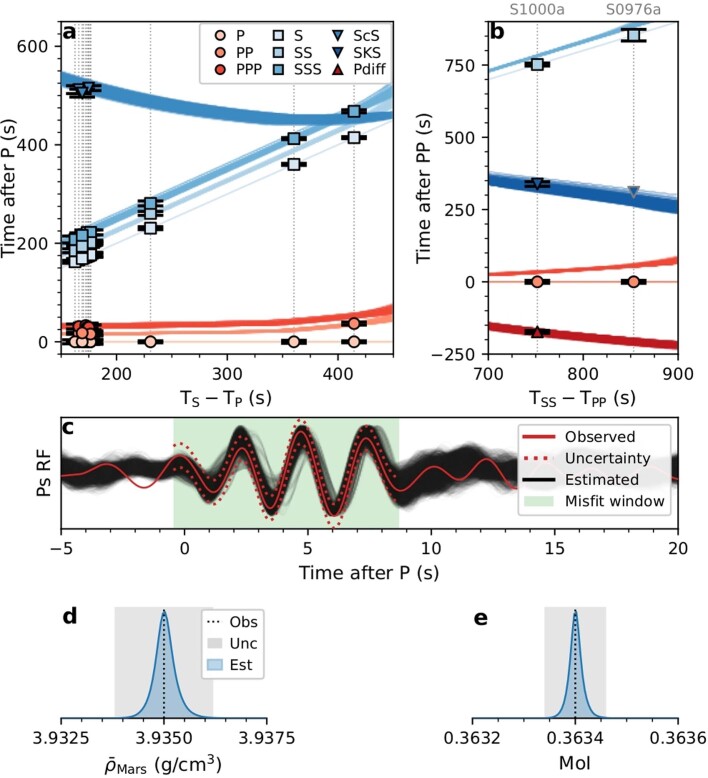
Seismic and geodetic data misfit. (a,b) Differential body wave travel time misfits for all sampled models shown in Fig. 2a. Blue and red lines denote differential travel times computed using the inverted models, and squares, circles, and triangles indicate the observations including error bars. Note that the SKS pick for S0976a is only predicted. Event picks are aligned by the observed S-P (panel A) and SS-PP (panel B) differential travel time. For the travel time calculations performed here, we always pick the first arrival. (c) P-to-s receiver function waveform (Ps RF) misfit. (d,e) Geodetic data misfit in the form of mean Martian density (${\bar{\rho }}_{{\rm{Mars}}}$) and mean normalised moment of inertia (MoI).

The liquid core of Mars, including the LSL, are considered to be homogeneous and well-mixed, and to compute seismic properties (density and P-wave velocity) we employ, in each region, an isentropic third-order Birch–Murnaghan EoS. The latter is parameterized using adiabatic bulk modulus (*K*_0*S*_), its pressure derivative (${K}_{0S}^{{\prime} }$) and molar density (*ρ*_0_) at the conditions of the solid mantle–LSL interface for the LSL and the LSL–liquid core interface in the case of the core, respectively. The temperature dependence of the EoS is implicit in the reference condition and the assumption of an adiabat, in spite of the EoS being isothermal. The parameters defining the structure of the crust, mantle and core of Mars are illustrated in Supplementary Fig. 5, while model parameters and prior model parameter ranges are summarized in Supplementary Table 1. To solve the inverse problem of jointly determining seismic velocity profiles and epicentral distances of seismic events, we employ the probabilistic approach of ref. ^64^. Assuming that data noise is uncorrelated and can be described by a Laplace distribution (L_1_-norm), the likelihood function takes the form

8 $${\mathcal{L}}({\bf{m}})\propto \exp \,\left(\,-\frac{1}{N}\mathop{\sum }\limits_{j}^{N}\frac{\parallel {{\bf{d}}}_{{\rm{obs}}}^{j}-{{\bf{d}}}_{{\rm{cal}}}^{j}\parallel }{{\sigma }_{j}}\right),$$

where **d**_obs_ and **d**_cal_ denote vectors of observed and synthetic differential travel times (compiled in Supplementary Table 2), stacked P-to-s RF waveform, mean mass (*M*) and mean moment of inertia (MoI), *σ*_*j*_ is the uncertainty on either of these datasets, and *N* is the total number of observations. Finally, to sample the posterior distribution, we employ the Metropolis sampling algorithm^64^ as a means of sampling solutions to the inverse problem. This algorithm ensures that models fitting data and that are consistent with prior information are sampled more frequently. The resultant data misfit is shown in Extended Data Fig. 6.

### Ab initio molecular dynamics simulations of silicate liquids

We conducted ab initio simulations using Vienna Ab initio Simulation Package^65^ based on the projector augmented wave method^66,67^. Two end-member compositions corresponding to a bulk Martian mantle^4^ and Shergottite Y980459 (ref. ^31^), respectively (Extended Data Table 2), were simulated in supercells consisting of 150 atoms, equivalent of the compositions Fe_6_Mg_27_Si_25_Ca_2_Al_2_O_88_ and Fe_8_Mg_16_Si_28_Ca_4_Al_4_O_90_, respectively. Atoms were initially randomly distributed and heated to 6,000 K for at least 10 ps to obtain silicate melt structure before being equilibrated at 2,100 K and 19 GPa, corresponding to the *P*–*T* conditions of the Martian CMB. The canonical ensemble (NVT) was used with a Nosé–Poincaré thermostat to control the temperature. Simulations were run for 10–15 ps with a time step of 1 fs to obtain the elastic properties (Extended Data Table 3). *P* and *T* were calculated from the time average. The details of the AIMD simulations and postprocessing can be found in ref. ^68^.

### Cosmochemical constraints

The higher volatility of C relative to S during cosmochemical processes (for example, ref. ^69^) means that S/C ratios in any potential chondritic building blocks should be minimum estimates for that of Mars. The S/C of the Martian core should reflect that in bulk Mars, owing to the moderately-to-highly siderophile behaviour of S and C during Martian core formation^70^. The Martian budget of the moderately volatile element Zn, with volatility comparable to S, is derived 9:1 from ordinary to carbonaceous chondrites^71^, suggesting a core S/C ratio of 9 ± 4, intermediate between CI chondrites (roughly 2) and ordinary chondrites (roughly 20). For 12 wt% S, this results in at most 2 wt% C in the Martian core. Such S- and C contents imply 1–2 wt% O in the Martian core^54^ (see Supplementary Information section 8 for more details).

## Online content

Any methods, additional references, Nature Portfolio reporting summaries, source data, extended data, supplementary information, acknowledgements, peer review information; details of author contributions and competing interests; and statements of data and code availability are available at 10.1038/s41586-023-06586-4.

### Supplementary information

Supplementary Information Supplementary Sections 1–9, equation (1), Figs. 1–15, Tables 1 and 2 and References.

## Figures and Tables

**Extended Data Table 1:**
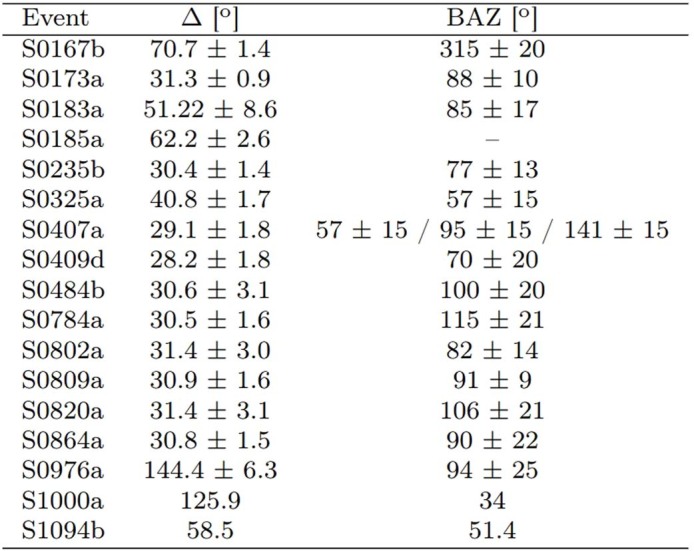
Marsquake and meteorite impact locations

Inverted epicentral distances ($\Delta$) for the seismic events considered in this study. Quoted values indicate mean and standard deviation from the sampled distributions. Events S1000a and S1094b represent meteoroid impacts whose distances are fixed after^33^. Back-azimuth (BAZ) determinations are from ref. ^76^ with the exception of the two impacts, which are from ref. ^33^. Multiple BAZ entries indicate the presence of several maxima in the respective BAZ distribution. Quoted values indicate mean and standard deviation.

**Extended Data Table 2:**
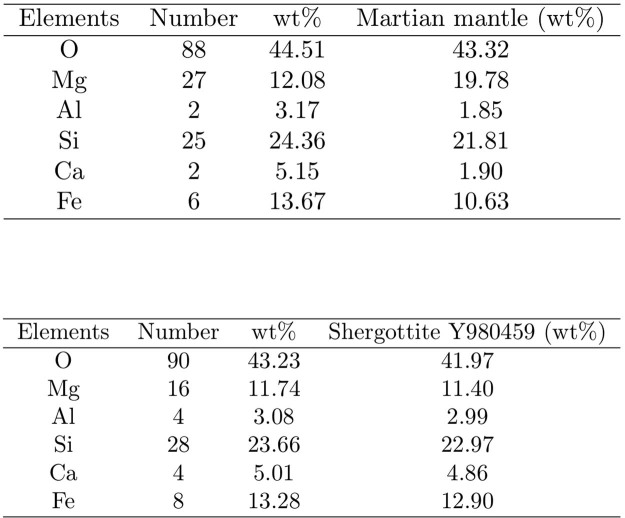
Silicate melt supercells modelled after a bulk Martian mantle and Shergottite Y980459 composition

Elemental abundances are given in number of atoms and wt%, and are compared with the Martian mantle composition of ref. ^4^ and the Shergottite composition of ref. ^31^, respectively.

**Extended Data Table 3:**
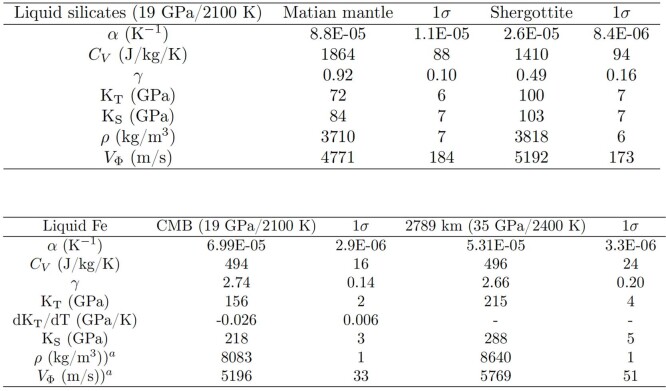
Calculated thermal expansion coefficient (*α*), specific heat capacity (*C*_*V*_), Grüneisen parameter (*γ*), isothermal bulk modulus (K_T_), dK_T_/dT, isentropic bulk modulus (K_S_), density (*ρ*) and bulk sound velocity (*V*_Φ_) of liquid silicates and Fe at the top of Mars’s core

Data for liquid iron are taken from ref. ^3^ for comparison. Uncertainties in density and velocity are obtained by equation-of-state fitting (Eq. (5)), followed by error propogation, which are smaller than the corresponding uncertainties in the mixing model.

## Data Availability

The InSight seismic event catalogue V14 (ref. ^59^) (comprising all events, including phase picks) and waveform data are available from the IRIS DMC, NASA-PDS, SEIS-InSight data portal and IPGP data centre. Interior Martian structure models are available in digital format from 10.18715/IPGP.2023.llxn7e6d.

## Code availability

Additional information related to this paper may be requested from the corresponding authors.
