# Supplementary material for: Evidence for a liquid silicate layer atop the Martian core
Source: Nature. 2023 Oct 25;622(7984):718–23. doi: 10.1038/s41586-023-06586-4 (PMC10600012; doi:10.1038/s41586-023-06586-4)
Supplement: Supplementary file 1 — Supplementary Sections 1–9, equation (1), Figs. 1–15, Tables 1 and 2 and References. [file 41586_2023_6586_MOESM1_ESM.pdf]

nature portfolio

https://doi.org/10.1038/s41586-023-06586-4

Supplementary information

Evidence for a liquid silicate layer atop the Martian core

In the format provided by the
authors and unedited

“Evidence for a liquid silicate layer atop the Martian core"

A. Khan[1,2*], D. Huang[1*], C. Durán[2], P.A. Sossi[1], D. Giardini[2] and M. Murakami[1]

[1]Institute of Geochemistry and Petrology, ETH Zürich, Switzerland.
[2]Institute of Geophysics, ETH Zürich, Switzerland.

*Corresponding author(s). E-mail(s): amir.khan@erdw.ethz.ch; dongyang.huang@erdw.ethz.ch;

Contents

1 SKS Identification **3**
1.1 S1000a 3
1.2 S0976a 5

2 Prior model parameter information **9**

3 Inversion results for a different mantle composition **11**

4 Differential body-wave travel time data set **12**

5 Seismic waveform modeling **14**

6 Observational evidence for a liquid silicate layer **16**
6.1 Diffracted P-waves 16
6.1.1 Filter banks 16
6.1.2 Polarisation analysis 17
6.1.3 Waveform matching 17
6.2 Core-interacting P-waves 18
6.3 Analysis of Sdiff 22

7 Core composition **23**

8 Geo- and cosmochemical constraints **24**

9 Estimating the Martian mantle liquidus **25**

1 SKS Identification

1.1 S1000a

Since a comprehensive description of the identification of PP- and SS-wave arrivals is given in [1], here we focus mainly on the identification of the SKS-wave arrival. Through the application of filter banks, polarized waveforms, and polarization analysis [2, 3], we refined the initial picks and considerably reduced the uncertainties of [4], allowing us to properly align the event and better constrain its location. Envelopes of the event with the PP- and SS-wave picks and their uncertainties represented as vertical lines and bars, respectively, are shown in Figure S1.

As the location of the event is well known [5], waveforms are rotated to and envelopes are shown in the Vertical-Radial-Transverse (ZRT) system. Gray curves in Figures S1A–C represent the time-domain envelopes of the band-pass filtered waveforms (0.2–0.5 Hz), while blue curves represent envelopes of the waveforms after applying a time-domain polarization filter [6]. Because body waves exhibit a high degree of linear polarization, the application of the polarization filter increases the signal-to-noise ratio of the linearly polarized part of the signal.

Figure S1D shows a three-component scalogram of the event computed as the absolute value of the continuous wavelet transform of the signal. This scalogram, built as the sum of the squared scalograms of each component, illustrates the change in frequency content of the signal with time. While the main PP- and SS-wave arrivals are discernible on account of the broad frequency content of the envelopes and scalogram, the identification of additional phases in the PP- and SS-wave coda is challenging, because of possible interference of phases and scattering signal after the PP-wave arrival, as illustrated in Figure S1. In addition, non-seismic signals in the form of large glitches are visible in Figure S1D, (smaller glitches are indicated by the orange box). Glitches, which are transient one-sided pulses often accompanied by high-frequency spikes [7], are mainly attributed to SEIS-internal stress relaxations or by tilts of the instrument [8], and contaminate the signal making the identification of seismic phases difficult.

To narrow the search for the SKS phase, we predict the arrival time of SKS by considering the Martian models of [3] and perturb the P-wave velocity in the core by ±15% to account for the lack of information on seismic core properties. For S1000a, the so-predicted time range is represented by the green shaded area in Figures S1A–C. While the time-domain envelopes of the non-polarized waveforms do not exhibit any particularly distinctive arrival within the expected time range, a peak is discernible around 340 s in the R component of the polarized waveform envelopes, where the SKS arrival is expected. This large-amplitude arrival, is strongly linearly polarized and consistent with synthetically-computed SKS waveform amplitudes that indicate a relatively

large-amplitude SKS phase (Figure S2). No other large-amplitude arrival compatible with the characteristics of SKS is discernible within the expected time window.

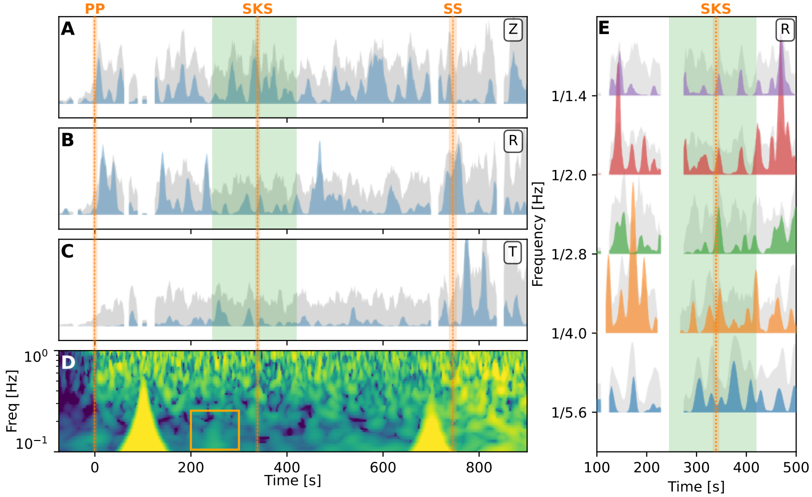

Fig. S1 Time-domain envelopes of band-pass filtered (0.2–0.5 Hz) polarized (blue) and non polarized (gray) waveforms of (A–C), three-component scalogram (D), and radial-component polarised (colour) and non-polarised time-domain waveforms and envelopes across different frequency bands (E) for event S1000a. Phase picks for S1000a and their uncertainties [1] are shown as vertical dashed lines and orange bars, respectively. Envelopes are masked where glitches occurred to avoid misinterpretation of phases. The vertical green band represents the expected arrival time range of SKS. Orange box in (D) indicates the presence of a small glitch. Envelopes are normalized for visual purposes. Time is relative to the arrival of the PP-wave.

As a means of validating the selected seismic phase, we filter the velocity traces in narrow frequency bands, allowing us to better visualize the partitioning of energy across different frequencies. We use a bandwidth half an octave wide around each central frequency, ranging from 1/5.7 to 1/1.4 Hz. The selected filter banks cover the frequency bands where the low-frequency energy of the event is most visible and is mainly devoid of glitch-, donk- or atmospheric-related artefacts [8]. Donks, interpreted as stress release from temperature cycling on the lander, are pulses of energy with a typical duration of a few seconds, and are generally observed at frequencies higher than 10 Hz [7]. Figure S1E shows the envelopes of the polarized and non-polarized R-component waveforms in a time window that includes the SKS-wave arrival. In the filter banks, we observe a strongly polarized arrival at frequencies ranging from 1/2.8 to 1/1.4 Hz that conforms with our established criteria for identifying seismic phases [3] and which we therefore pick as the SKS phase.

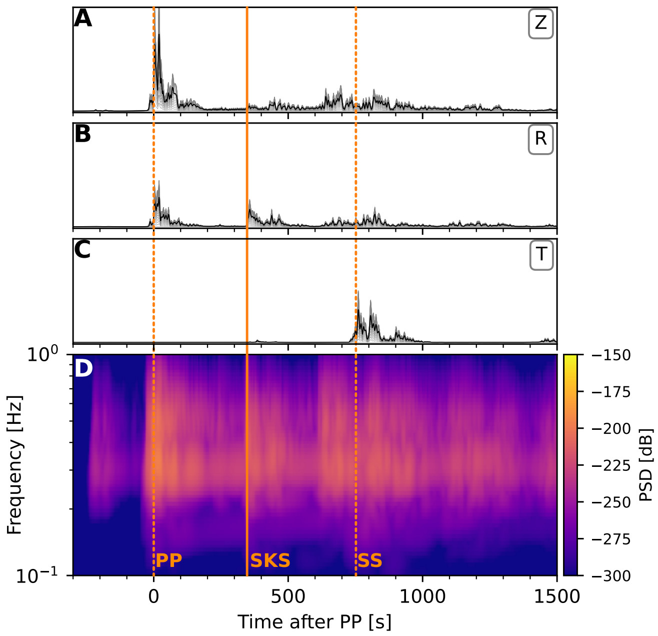

Fig. S2 Time-domain envelopes (A–C) and three-component scalograms (D) for a synthetic event with a differential travel time T_{SS}–T_{PP} matching the observation for S1000a. To represent an impact, we compute synthetic waveforms simulating an inclined force at the surface for a range of angles, including vertical force. Their time-domain envelopes are shown in gray, while a mean representative envelope is shown in black. Arrival times for PP and SS computed using ray theory are shown as dotted vertical lines, while the arrival time for SKS is shown by a solid vertical line.

1.2 S0976a

Analogous to the analysis presented for event S1000a, Figure S3 shows envelopes of polarized (blue) and non-polarized (gray) waveforms in the Vertical-North-East (ZNE) system (Figure S3A–C) and three-component scalogram (Figure S3D) for event S0976a. While an estimate of the back-azimuth for event S0976a based on the observed particle motion of the PP-wave arrival is available [9], the uncertainty is considerable (±25°).

In contrast to S01000a, S0976a has a much more impulsive PP-wave arrival, allowing us to easily pick it in the band-pass filtered (0.1–0.5 Hz) polarized and non-polarized waveforms. In addition, its arrival is clearly discernible in the scalogram, characterized by a considerable increase of energy. For the SS-wave arrival, due to the presence of a small glitch which affects the waveforms, we mainly rely on the scalogram to identify the energy onset. Both, PP- and SS-wave picks are represented as vertical lines in Figure S3. While the increase of energy is clear in the scalogram for both PP- and SS-waves, the picks also coincide with the break of the slope in the Z-component envelopes for PP and the horizontal components for SS. However, due to the presence of several glitches close to the PP- and SS-wave arrivals and the windy conditions that

are present throughout the event [9], we assign larger uncertainties to both picks (represented by the vertical bars in Figure S3) in comparison to event S1000a.

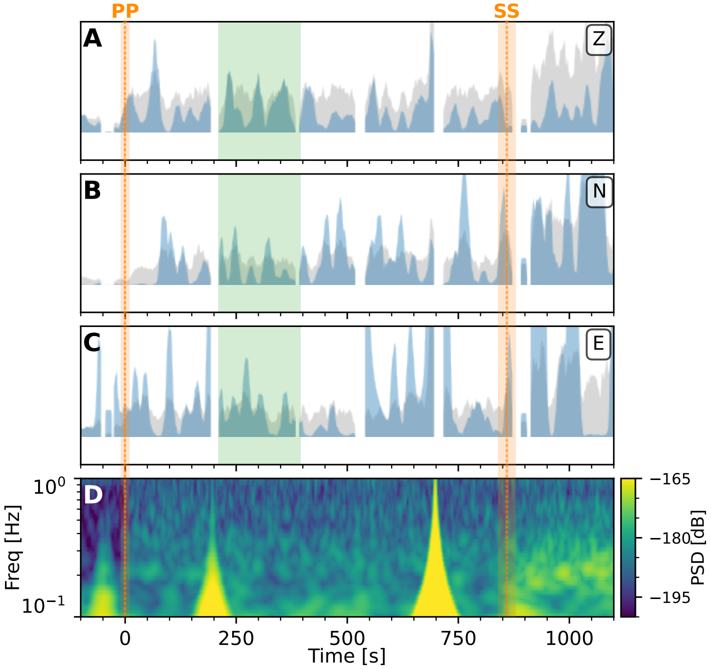

Fig. S3 Time domain envelopes of band-pass filtered (0.2–0.5 Hz) polarized (blue) and non polarized (gray) waveforms of (A–C) and three-component scalogram (D) of event S0976a. Phase picks for S0976a and their uncertainties [1] are shown as vertical solid lines and orange bars, respectively. Envelopes are masked where glitches occurred to avoid misinterpretation of phases. Green band represents range for expected arrival of SKS based on inverted models where the core velocities were perturbed ±15%.

Because S0976a has not been reliably located, the prediction of a potential SKS phase relies on the alignment of the event through PP- and SS-wave arrival times. We follow the procedure for S1000a and compute a range of arrival times for SKS to narrow down the possible arrival time interval of SKS. In comparison to S1000a, where a single strong SKS candidate is apparent, for S0976a a series of similar-amplitude and strongly linearly-polarized arrivals in the predicted time window for SKS are present. The lack of a clear candidate for SKS could be due to 1) lack of an accurate location that would enable a rotation of the traces into the ZRT system and 2) the generally noisy (wind) conditions that characterise the event [9].

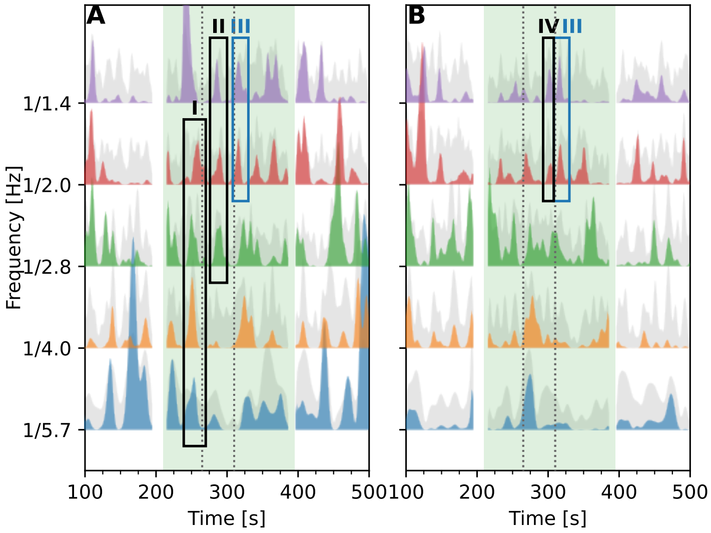

Fig. S4 Time-domain envelopes across different frequency bands (filter banks) of polarized (color) and non polarized (gray) waveforms in the North (A) and East (B) components. Green-shaded area represents predicted range for SKS arrival based on inverted models and alignment of the differential travel time $T_{SS} - T_{PP}$. Vertical dotted lines indicate predicted range for SKS arrival based on inverted models [3] that fit the SKS travel time of S1000a. Boxes indicate potential SKS-wave arrivals for each component. Amplitudes of traces and envelopes are normalized by their maxima and scaled for better visualisation. Time is relative to arrival of PP seismic energy in the time-domain waveforms.

The lack of a precise location requires analysis of both North and East components. Time-domain envelopes across different frequency bands are shown in Figure S4 for both horizontal components. The time window during which SKS is expected is indicated by the green-shaded area in Figure S4, and several linearly-polarized peaks are present that are labeled from I–IV. Of these, we can rule out I because it would correspond to an SKS arrival that is too early relative to what we would expect based on S1000a (this range is represented by dotted vertical lines). This leaves arrivals II, III, and IV. Of these, arrival II ($\sim$285 s) is only seen on the North component but present across the same frequencies (1/2.8–1/1.4 Hz) as S1000a, whereas arrival III ($\sim$310 s) appears on both components and at the same frequencies (1/2 and 1/1.4 Hz). Because of this fundamental uncertainty in the SKS arrival, we abstain from picking the latter for event S0976a.

In [10], several independent methods were employed to pick SKS for S0976a based on either waveform or cross correlation methods. The SKS-PP travel time spread for the five methods covers the range from 297.4 s to 311 s with two picks in the lower range (297.4 s and 298.3 s) and three picks toward the upper range (309 s, 310 s, and 311 s). Irving et al. resorted to averaging all the picks as a means of producing a representative SKS pick (303.9 s) and used the standard deviation of the five picks as indicative of pick uncertainty

(7.1 s). This final pick, however, is not consistent with all of the individual picks. Clearly some of the picks represent outliers. In contrast, here we rely on a single processing/picking method for consistency, yet we refrain from picking SKS for S0976a because of the inherent difficulty in making a consistent pick.

2 Prior model parameter information

Model parameters are illustrated in Figure S5, while model parameters and prior model parameter ranges are summarized in Table S1 below. The prior ranges follow our earlier studies [e.g., 2, 3, 11, 12] and cover wide model parameter ranges.

Table S1 Overview of model parameters, their quantity, prior model ranges and probability distributions employed in the geophysical parameterization

Description	Param.	Quantity	Value/range	Distribution
**Crust**				
S-wave velocity	V_S^i	3	1.5–4.2 km/s	uniform[a]
P-wave velocity	V_P^i	-	$\alpha \cdot V_S^i$	uniform[b,c]
V_P/V_S scaling	α	1	1.65–1.85	uniform
Moho depth	Z_c	1	20–60 km	uniform
Layers thickness	ΔZ_i	3	1–50 km	uniform
Surface temperature	T_{surf}	1	273 K	fixed
**Mantle**				
Lithospheric temperature	T_{lit}	1	1273–1873 K	uniform
Lithospheric depth	Z_{lit}	1	100–600 km	uniform
Mantle composition	X_m	1	[12]	fixed
**Liquid Silicate Layer**				
Layer thickness	ΔZ	1	20–300 km	uniform
Adiabatic bulk modulus	K_{0S}	1	40–200 GPa	uniform
Pressure-derivative of bulk modulus	K'_{0S}	1	3–8	uniform
Molar density	ρ_0	1	3–5 g/cm^3	uniform[d]
**Core**				
Core radius	R_{core}	1	1300–2000 km	uniform
Adiabatic bulk modulus	K_{0S}	1	40–250 GPa	uniform
Pressure-derivative of bulk modulus	K'_{0S}	1	3–8	uniform
Molar density	ρ_0	1	4–8 g/cm^3	uniform[e]
**Marsquakes**				
Epicentral distance (S1000a)	Δ	1	125.9	fixed
Epicentral distance (S1094b)	Δ'	1	59.9	fixed
Epicentral distance (LF events)	Δ_{LF}	15	0°–180°	uniform
Depth	h	17	0–100 km	[3]

[a] V_S increases with depth.
[b] V_P increases with depth.
[c] V_P always larger than V_S.
[d] ρ_0 of the liquid silicate layer is always larger than or equal to ρ of the solid mantle layer immediately above.
[e] ρ_0 of the core is always larger than or equal to ρ_0 of the liquid silicate layer.

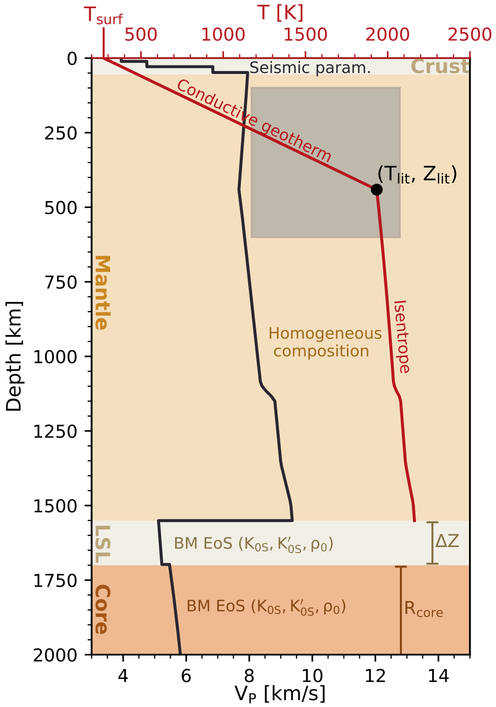

Fig. S5 Thermo-chemical and seismic model parameterisation (crust). An example areotherm is shown in red and defined using the parameters surface temperature T_{surf} (fixed), temperature at the base of the lithosphere T_{lit}, and lithospheric thickness Z_{lit}. Prior ranges on T_{lit} and Z_{lit} are indicated by the grey box. Temperatures below the lithosphere are computed from the entropy of the lithology at the base of the lithosphere. Mantle composition is assumed to be homoegenous, indicated by the single-coloured region from the base of the crust to the top of the liquid silicate layer (LSL). All model parameters and prior ranges are defined in Table S1. For illustration, a self-consistently-computed shear-wave velocity profile for the red areotherm is shown in black (for the composition of [13]). The LSL and liquid core are described using separate 3rd-order Burch-Murnaghan (BM) EoSs and parameterised in terms of adiabatic bulk modulus K_{0S}, its pressure derivative K'_{0S}, and molar density ρ_0, referenced at the conditions of the solid mantle-LSL interface for the LSL and LSL-liquid core interface for the core, respectively. The thickness of the LSL is determined by ΔZ, whereas core radius is defined by R_{core}.

3 Inversion results for a different mantle composition

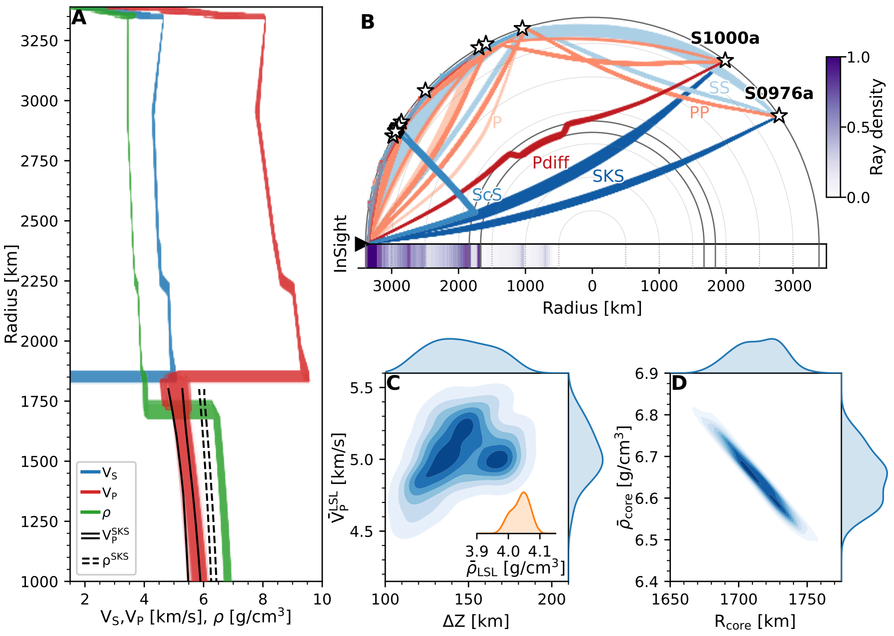

Fig. S6 Summary of Mars's interior structure. (A) Inverted S- and P-wave velocity and density profiles. For comparison, black solid and dashed lines in (A) represent the range of core profiles determined previously from seismic core-transiting (SKS) data [10]. (B) Body wave ray path geometry for all events (labeled with stars) considered in this study. Colorbar denotes ray path density, i.e., the number of rays passing through a given area, based on the inverted models shown in panel (A), which explains the diffuseness of the ray paths and source locations. The horizontal column underneath "InSight" is the radial sensitivity and computed as the integrated ray path density with epicentral distance. (C) Inverted liquid silicate layer (LSL) properties in the form of mean density ($\bar{\rho}_{LSL}$), mean P-wave velocity ($\bar{V}_P^{LSL}$), and thickness (ΔZ). (D) Inverted core properties in the form of mean density ($\bar{\rho}_{core}$) and core radius (R_{core}).

4 Differential body-wave travel time data set

Table S2 Summary of body-wave travel time picks used in the present study based on the present work and [1, 3, 10]. T_0 represents the P-wave arrival time in UTC, except for events S0976a and S1000a, for which T_0 indicates the PP-wave arrival time. Tabulated times are expressed in seconds after T_0. The SKS-wave pick arrival of S0976a based on [10] is uncertain as discussed in section 1 and is not employed in the present inversion.

Event	T_0	T_{pP}	T_{PP}	T_{PPP}	T_S	T_{sS}	T_{SS}	T_{SSS}	T_{PS}	T_{ScS}	T_{SKS}	T^{LSL}_{Pdiff}	T^{CMB}_{Pdiff}	$T_{\mathrm{Pdiff\hat{}LSL\ Pdiff}}$
S0167b	2019-05-17T19:31:35 (±2)	–	37.0±3	–	414.5±2	–	–	468.0±3	–	–	–	–	–	–
S0173a	2019-05-23T02:22:57 (±3)	11.1±3	–	–	174.8±2	184.8±3	197.9±2	–	–	515.0±5	–	–	–	–
S0183a	2019-06-03T02:27:45 (±3)	–	24.5±4	43.0±7	–	–	–	–	–	–	–	–	–	–
S0185a	2019-06-05T02:14:09 (±3)	–	–	–	360.2±2	379.5±4	–	412.5±6	–	–	–	–	–	–
S0235b	2019-07-26T12:19:17 (±2)	–	17.4±2	31.1±7	166.0±3	178.7±3	193.1±3	–	168.4±2	512.0±8	–	–	–	–
S0325a	2019-10-26T06:58:54 (±2)	11.3±3	–	–	230.7±3	–	260.7±4	281.0±6	–	–	–	–	–	–
S0407a	2020-01-19T09:57:41 (±3)	–	17.8±5	33.0±7	172.2±2	183.4±3	196.4±5	–	–	–	–	–	–	–
S0409d	2020-01-21T11:31:22 (±4)	–	–	–	162.5±3	–	184.9±4	207.7±6	–	–	–	–	–	–
S0484b	2020-04-07T08:52:34 (±3)	–	18.1±5	–	170.2±1	184.0±3	196.8±3	–	–	–	–	–	–	–
S0784a	2021-02-09T12:16:13 (±5)	–	15.2±4	29.5±7	173.0±4	182.2±4	196.9±5	221.9±6	178.7±2	–	–	–	–	–
S0802a	2021-02-28T06:11:03 (±4)	–	17.3±5	–	176.3±5	–	201.5±5	222.1±6	–	–	–	–	–	–
S0809a	2021-03-07T11:13:12 (±3)	–	15.5±5	29.3±7	175.0±1	–	197.5±3	–	184.0±2	–	–	–	–	–
S0820a	2021-03-18T14:55:20 (±3)	–	15.7±5	–	176.5±2	186.2±3	201.4±5	–	–	–	–	–	–	–
S0864a	2021-05-02T01:01:08 (±5)	–	18.0±5	–	169.0±3	181.2±4	194.0±3	216.1±6	–	505.0±8	–	–	–	–
S0976a	2021-08-25T03:49:01 (±8)	–	–	–	–	–	859.0±20	–	–	–	(*310±8**)	–	–	–
S1000a	2021-09-18T18:01:57 (±5)	–	–	–	–	–	746.0±8	–	–	–	339±5	-235±5	-177±5	-113±5
S1094b	2021-12-24T22:45:09 (±1)	–	–	–	342.8±20	–	–	–	–	–	–	–	–	–

*Uncertain pick

5 Seismic waveform modeling

We use the spectral element code AxiSEM [14] to model high-frequency waveforms that match the frequency content of the data (up to 1 Hz) assuming an inclined (45°) source. Figures S7–S8 show synthetic waveform sections for a melt-layer model obtained here (Figure 2A) and a Martian model from [11].

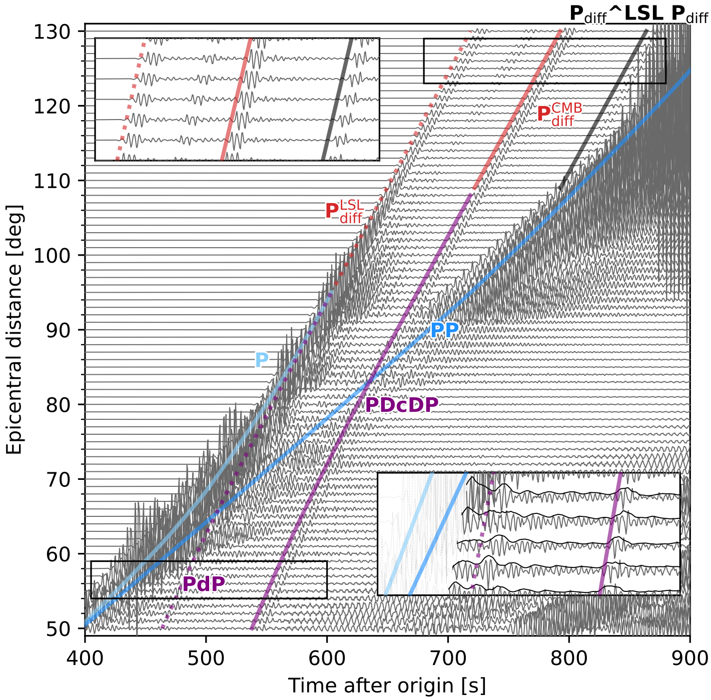

Fig. S7 Synthetic section of vertical component waveforms for epicentral distances ranging from 50 to 130°. Waveforms are computed employing the spectral element code AxiSEM [14] assuming an inclined (45°) source. Main body-wave arrivals (P and PP) and arrivals of phases that interact with the liquid silicate layer (LSL) are indicated by dotted and solid curves across the section. Inset at the top provides a zoom into the predicted diffracted P-waves from the mantle-LSL interface (P_{diff}^{LSL}) and the LSL-liquid core interface (P_{diff}^{CMB}) for epicentral distances similar to that of S1000a (∼ 126°). Inset at the bottom provides a zoom into the core-reflected P-waves from the top of the LSL (PdP) and the liquid core (PDcDP) for epicentral distances similar to that of S1094b (∼ 59°). Black dashed lines demarcate both areas for which zoom in insets are produced.

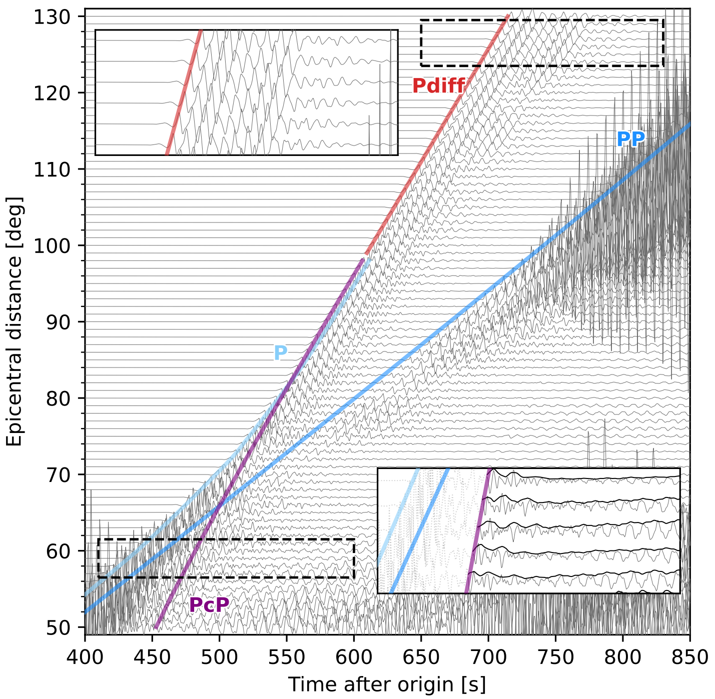

Fig. S8 Synthetic section of vertical component waveforms for epicentral distances ranging from 50 to 130° employing an earlier Martian model [11]. Waveforms are computed employing the spectral element code AxiSEM [14] assuming an inclined (45°) source. Main body-wave arrivals (P and PP) and arrivals of phases that interact with the core-mantle boundary (CMB) are indicated by solid curves across the section. Inset at the top provides a zoom into the predicted diffracted P-waves (Pdiff) from the CMB interface for epicentral distances similar to that of S1000a (∼ 126°). Inset at the bottom provides a zoom into the core-reflected P-waves from the top of the core (PcP) for epicentral distances similar to that of S1094b (∼ 59°). Black dashed lines demarcate both areas for which zoom in insets are produced.

6 Observational evidence for a liquid silicate layer

Generally, marsquake seismograms are characterized by scattering coda that complicates identification of seismic body wave phases needed for structural interpretation [2, 3, 15, 16]. As a consequence, seismic arrivals, with the exception of the direct P- and S-waves, are not immediately identifiable in the time series. Instead, a number of processing steps have been developed specifically for the purpose of detecting seismic phase arrivals, revolving around the use of narrow-band-filtered time-domain polarized and unpolarized envelopes and waveforms labelled filter banks [2, 3]. This increases the signal-to-ratio of the linearly-polarized part of the signal that involves body-wave phases [6]. Filtered polarized vertical-component traces and their time-domain envelopes for events S1000a and S1094b are shown in Figure S9. The polarized traces, in comparison to the original waveforms (not shown) exhibit packages of energy that are associated with body wave arrivals and allows for improved understanding of frequency-dependent energy partitioning.

6.1 Diffracted P-waves

6.1.1 Filter banks

We filtered the waveform traces (velocity) in narrow frequency bands using a bandwidth half an octave wide around each central frequency, ranging from 1/5 to 1 Hz, and computed their envelopes. The selected filter banks cover the frequency bands where the low-frequency energy of the event is most visible and is mostly devoid of glitch-, donk- or atmospheric-related artefacts [17]. For event S1000a, the main diffracted P-wave arrival ($\mathrm{P}_{\mathrm{diff}}^{\mathrm{CMB}}$) is discernible across four filter banks from 1/1.4 Hz to 1/4 Hz, whereas the lower-amplitude P-wave that is diffracted along the mantle-liquid silicate layer interface ($\mathrm{P}_{\mathrm{diff}}^{\mathrm{LSL}}$) and the reverberation ($\mathrm{P}_{\mathrm{diff}}$^LSL $\mathrm{P}_{\mathrm{diff}}$) are visible across the frequency bands from 1/1.4 Hz to 1/2.8 Hz (Figure S9C). The onsets of the individual energy packages in the filter banks coincide with the main arrivals that are visible in the polarized waveform and envelope shown in Figure S9B. Based on the identification procedure of body-wave arrivals described in [3], we identify discrete energy onsets as seismic phases from the filter banks and the polarized and non-polarized traces/envelopes. To qualify as seismic phases, arrivals must be present across different frequency bands and in both, non-polarized and polarized traces/envelopes. None of the other arrivals exhibit these characteristics. Following this, the identified arrivals are selected as possible $\mathrm{P}_{\mathrm{diff}}^{\mathrm{LSL}}$ and $\mathrm{P}_{\mathrm{diff}}$^LSL $\mathrm{P}_{\mathrm{diff}}$ picks as indicated in Figure S9. Note that $\mathrm{P}_{\mathrm{diff}}^{\mathrm{CMB}}$ has been identified by [1]. The vertical red bar in Figure S9B,C indicate the differential travel time ranges relative to the main PP-wave arrival (for S1000a) computed using the inverted models shown in the main manuscript in Figure 2A. The identified $\mathrm{P}_{\mathrm{diff}}^{\mathrm{LSL}}$ arrival and the predictions based on the inverted models are seen to be in good agreement.

6.1.2 Polarisation analysis

To provide an independent verification of the selected arrivals, we follow [2, 3] and consider the particle motion (polarization). A scalogram of the event, computed as the absolute value of the continuous wavelet transform of the signal, is shown in Figure S10A and illustrates how the frequency content of the signal changes with time. Energy is seen to increase over the background noise in the frequency range and starting in the time window for which the inverted models predict the arrival of P_{diff}^{LSL} (delimited by dashed lines). As rectilinear polarization with consistent particle motion is expected for the diffracted P-waves, we compute the polarization attributes of the three-component seismic data and infer the instantaneous back-azimuth following [18]. To represent changes in azimuth with the arrival of seismic phases, we compute normalized azimuthal probability density curves in the frequency band 0.2–0.7 Hz considering 10-s long time windows. These are shown in Figure S10B. Until the arrival of the P_{diff}^{LSL} wave, no consistent azimuth is observed. With the arrival of P_{diff}^{LSL}, however, particle motion appears to be consistent with the azimuth based on the imaged location (indicated by the horizontal red line). Following the arrival of P_{diff}^{LSL}, the azimuth generally exhibits a consistent pattern around 30° across the Pdiff wavetrain.

6.1.3 Waveform matching

Since P_{diff}^{LSL}, P_{diff}^{CMB}, and $P_{diff}\hat{}LSL\,P_{diff}$, follow approximately similar paths, their waveforms are expected to be similar. This provides another means of verifying our selected arrivals. We filter the vertical- and radial-component waveforms (0.2-–0.7 Hz) and consider a 10-s long window that includes the observed P_{diff}^{CMB} arrival to build a template trace. The vertical-component event and template trace are shown in Figure S10C in dark gray and red, respectively. Following this, we cross-correlate both traces considering lags every 0.05 s, and compute at each lag a correlation coefficient CC_Z and CC_R for the vertical and radial component, respectively, defined by the mean of the cross-correlation function. The similarity-coefficient employed to measure resemblance between the two diffracted P-wave arrivals is computed as the root-sum-square of the cross-correlation coefficients for both components ($\sqrt{CC_Z{}^2 + CC_R{}^2}$). Figure S10D shows the temporal evolution of the similarity coefficient (gray) and the threshold (red horizontal line) employed to detect waveforms that match the template trace. The analysis indicates three prominent peaks in the similarity coefficient that are above our defined threshold that are indicated by the vertical coloured lines in Figure S10C and are seen to coincide with the arrival of P_{diff}^{LSL} and $P_{diff}\hat{}LSL\,P_{diff}$, in conformity with the synthetic waveform predictions (Figure S2). Based on the similarity in moveout, waveform, and differential travel time between P_{diff}^{CMB} and P_{diff}^{LSL}, on the one hand, and $P_{diff}\hat{}LSL\,P_{diff}$ and P_{diff}^{CMB}, on the other hand, the third phase represents a reverberating P_{diff}^{CMB} in the liquid silicate layer.

6.2 Core-interacting P-waves

For event S1094b (Figure S9C), we identify packages of energy in the envelopes and waveforms across the main frequency bands (1/1.4 Hz and 1/2 Hz), where seismic arrivals from low-frequency events are expected. Note that for this particular event the main P-wave arrival can only be identified above 1 Hz. The vertical orange/blue bars indicate the differential travel time ranges relative to the main P-wave arrival computed using the inverted models shown in the main manuscript in Figure 2A. However, because the impact event S1094b is overall noisy and given the location of PdP and PDcDP in the PP-wave coda, identifying these arrivals is very challenging even with the filter banks. For these reasons polarization and waveform matching analysis as performed for S1000a is equally difficult.

Finally, Figure S11 shows a waveform section with the travel time curves for PdP and PDcDP superposed. This figure indicates that for S-P differential travel times below 200 s ($\Delta < 35°$), PdP and PDcDP will be located in the S-wave coda and thus largely unidentifiable. According to Figure S11, the optimal epicentral distance range for observation of PdP and PDcDP is $\sim$35°–60°, i.e., S-P differential travel times around 225–350 s.

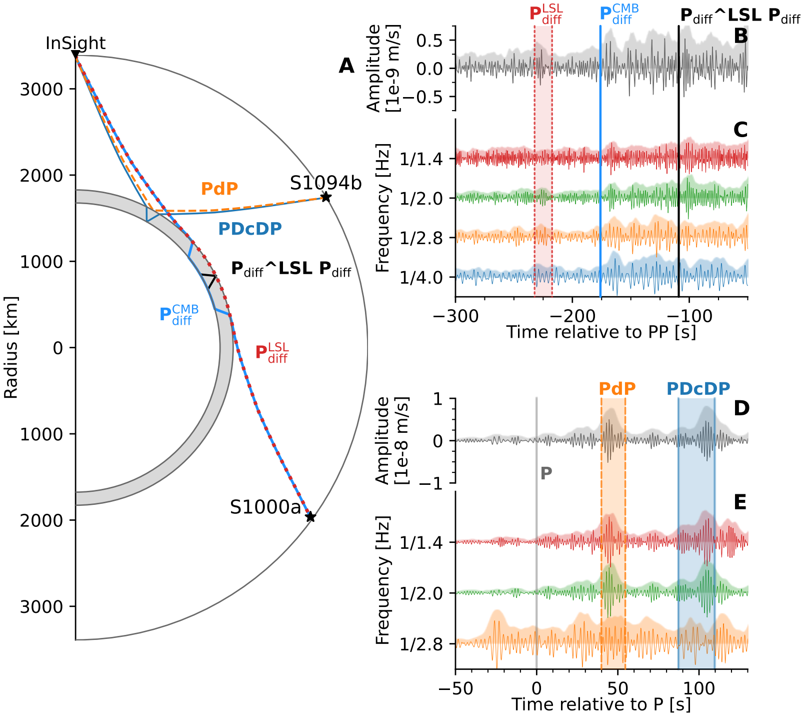

Fig. S9 Body-wave ray path geometry of seismic phases that interact with the liquid silicate layer (LSL) (A) and analysis of vertical-component waveforms of events S1094b (B–C) and S1000a (D–E). Panel (A) shows the predicted ray paths of seismic phases that provide additional constraints on the LSL: diffracted P-waves from the mantle-LSL interface (P_{diff}^{LSL}) and the LSL-liquid core interface (P_{diff}^{CMB}), including a diffracted P wave reverberating in the LSL ($P_{diff}\hat{}LSL\ P_{diff}$); and core-reflected P-waves from the top of the LSL (PdP) and the liquid core (PDcDP). The source locations of the high-quality events S1094b ($\Delta \sim 59^o$) and S1000a ($\Delta \sim 126^o$) employed to predict the ray paths are represented by black stars. Panel (B) shows the vertical-component waveform (dark gray) and envelope (light gray) for event S1000a (filtered between 0.2–0.7 Hz). The arrivals of P_{diff}^{LSL}, P_{diff}^{CMB}, and $P_{diff}\hat{}LSL\ P_{diff}$ are indicated. Also shown is the time window where the inverted models (Figure 2A) predict the arrival of P_{diff}^{LSL} (red vertical bar). (C) Waveform traces (dark color) and their time-domain envelopes (light color) filtered across narrow frequency bands (filter banks). (D) polarized vertical-component waveform (dark gray) and envelope (light gray) for event S1094b (filtered between 0.2–0.7 Hz) in the time window where the inverted models predict the arrivals of PdP and PDcDP (orange and blue vertical bars). Accompanying filter banks are illustrated in panel (E). Each trace is filtered using a bandwidth half an octave wide around each indicated central frequency. Amplitudes of traces and envelopes in (C) and (E) are normalized by their maxima for better visualization.

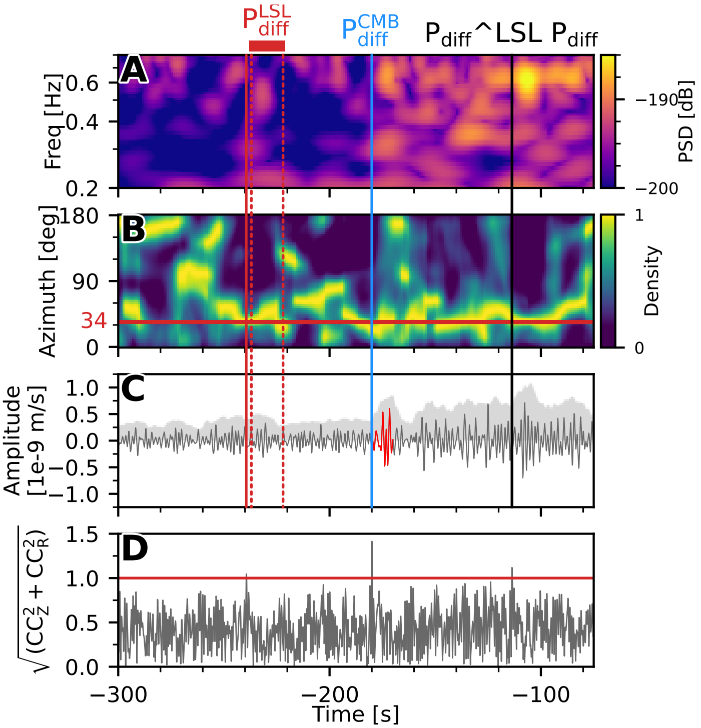

Fig. S10 Analysis of event S1000a in a time window showing the arrivals of the diffracted P-waves. The three-component scalogram of event S1000a and the temporal change in azimuthal density in the 0.2–0.7 Hz frequency band are shown in panels (A) and (B), respectively. For comparison, the horizontal red line in (B) represents the azimuth based on the imaged location. Panel (C) shows the vertical-component waveform (dark gray) and envelope (light gray) for event S1000a (filtered between 0.2–0.7 Hz). The vertical-component template trace employed for waveform matching is superimposed in red. Solid coloured lines in (A–C) indicate the times at which the similarity between the template and event trace is higher than 1. The similarity between the event trace and template trace is computed as the root sum squared of the cross-correlation coefficients obtained for the vertical (CC_Z) and radial (CC_R) components, and is shown in panel (D). Horizontal red line in (D) indicates the threshold employed for the waveform matching detections. Horizontal red bar on top and vertical red dotted lines in panels A–C represent the predicted arrivals of P_{diff}^{LSL} based on the inverted models.

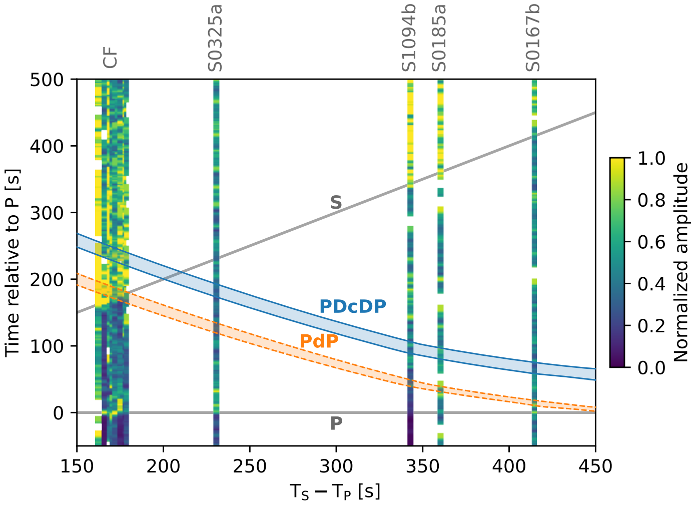

Fig. S11 Waveform section showing bandpass filtered (0.2–0.8 Hz) vertical-component envelopes (color) and predicted travel time curves for the direct P- and S-waves and interface- and core-reflected P-waves (PdP and PDcDP) based on the inverted seismic models shown in Figure 2A. Each trace (envelope) is normalised to the S-wave. CF designates the differential travel time distance range for the low frequency events that cluster around Cerberus Fossae, while the remaining events appear to be unrelated to any surface tectonic expression [18].

6.3 Analysis of Sdiff

As in the case of the diffracted P-wave (SM section 6.1), the inverted models presented here should give rise to diffracted S-waves along the mantle-LSL and LSL-liquid core interfaces. Following the analysis of [1], we first compute synthetic seismograms to analyze the characteristics that can be expected for Sdiff waves. As before, we employ the spectral element code AxiSEM [14] to model high-frequency waveforms that match the frequency content of the data (up to 1 Hz) assuming, as before, an inclined (45°) force. From the computed seismogram, we calculate the time-domain envelope by taking the square root of the summed squared amplitudes of the trace and its Hilbert-transform in 5 s long time windows to better depict the arrivals of energy packages. The time-domain envelopes for the vertical, radial, and transverse components are shown in gray in Figure S12A. While a robust SKS-wave arrival is observed in the radial component, Sdiff, which is expected to arrive with transverse polarization, is barely visible. In line herewith, Sdiff is unindentifiable in the observed seismograms (Figure S5C).

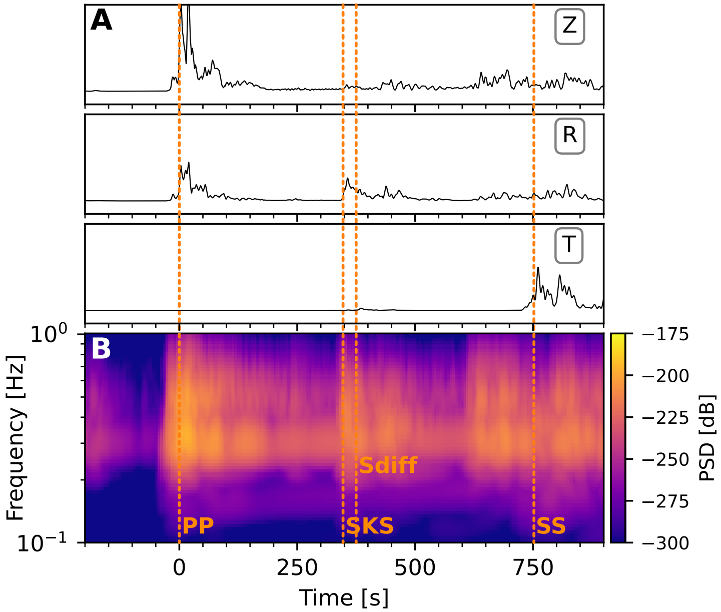

Fig. S12 Time-domain envelopes (A) and three-component scalograms (B) for a synthetic event with a differential travel time of $T_{SS} - T_{PP}$ matching the observation of S1000a. Synthetic waveforms are computed employing an inclined (45°) force as source. Arrival times for PP, SKS, Sdiff, and SS computed using ray theory are shown as dotted vertical lines. Time is relative to the theoretical PP-wave arrival.

7 Core composition

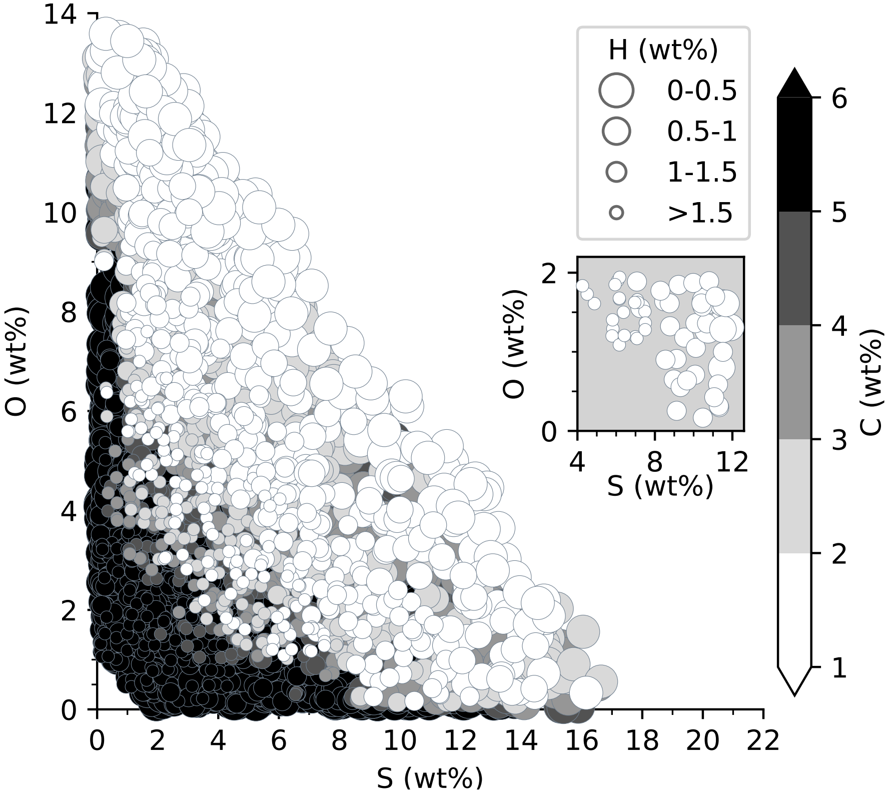

Fig. S13 Mars's core composition and light element budget. Each gray-shaded circle represents a Martian core composition in the liquid Fe-Ni-X (X=S,C,O,H) senary system, which has been obtained by matching density of the Fe-Ni-X mixtures based on the *ab initio* molecular dynamics (AIMD) simulations with the inverted seismic core profiles (corresponding to the light blue profiles in Figure 4 in the main text). The inset shows the subset of core compositions that remain after application of geo- and cosmochemical constraints (see Methods and SM section 8).

8 Geo- and cosmochemical constraints

Experimental and cosmochemical arguments can place further constraints on the composition of the Martian core. The solubility of C is 4–4.5 wt% in S-rich (10 wt%) Fe alloy near the conditions of Martian core formation, $\sim$13 GPa and 2073–2273 K [19]. As such, model compositions that exceed 4.5 wt% C are not realistic candidates for the Martian core. The light element content may be further restricted by considering the compositional range of potential Martian precursors. Of the extant set of meteorites, the chondritic meteorites, by virtue of their high volatile contents relative to their achondritic counterparts [20], are the most likely sources of S, C, O, and H. Mass-independent stable isotope anomalies of refractory lithophile elements and O in Mars indicate a strong affinity for ordinary chondrites with a lesser contribution from carbonaceous chondrites [21–23]. Given that S is less volatile than C during cosmochemical processes [e.g. 24], the S/C ratios in any potential chondritic building blocks should be minimum estimates for that of Mars. Both S and C are moderately- to highly siderophile during Martian core formation [19] such that $>$99.5% of the total Martian budget of S is expected to reside in the core, increasing to $>$99.9% for C. Thus, the S/C ratio of the Martian core is comparable to that of bulk Mars, and hence can be used to compare with those of chondritic meteorites. The data of [20] indicate that the S/C ratio ranges from $\sim$2 (CI) to $\sim$20 (H, L, LL). Using the abundances of moderately volatile lithophile elements to fix the bulk S content of Mars [12], the maximum S content of the Martian core, given its smaller mass fraction (21 ± 0.5%), is 12 wt%. Chondritic constraints then place C at between 0.6 wt% and 6 wt%. The upper end of this range is unfeasible because i) it exceeds C saturation and ii) CI chondrites alone are likely not viable precursors for Mars. Based on previously-constrained OC:CC ratios of 9:1 for the moderately volatile element Zn [25], which has a similar volatility to S, we consider the S/C ratio of bulk Mars to be $\sim$6, but with considerable uncertainty. This would translate into no more than 2 wt% C in the Martian core. However, C-rich compositions around 3–4 wt% C cannot be excluded cosmochemically. Provided that an estimate for S near 9–12 wt% holds for the Martian core, then, together with the temperature of equilibration and the FeO content of the mantle, the O content of the Martian core is readily calculated. The addition of S to Fe-rich alloy causes a strong decrease in the activity coefficient of O, stabilising O in the metal as S increases [26, 27]. At 2100 K and a mantle FeO content of 13.7 wt% from [12], the expression of [27] returns 1–2 wt% O in the Martian core.

9 Estimating the Martian mantle liquidus

In order to estimate the liquidus temperature of an hypothetical liquid layer at the base of the Martian mantle, the crysoscopic equation [28] is employed:

$$T_{liq} = -\left[\frac{R\ln(x/x_0)}{\Delta H_{fus}} - \frac{1}{T_0}\right]^{-1} \tag{1}$$

The form shown above comes from integration of the van't Hoff equation from a reference temperature T_0 and composition x_0 (here, $x = \mathrm{Mg}/[\mathrm{Mg}+\mathrm{Fe}^{2+}]$), to the temperature, T and composition, x of interest (R is the gas constant). For the simple binary system approximating that of the Martian mantle, which, like that of the Earth, is dominated by olivine [e.g., 12, 29], ΔH_{fus} can be estimated by that of forsterite/fayalite; that is, the liquidus temperature relates to that of the reaction: olivine(s) = olivine(l).

The value of ΔH_{fus} has been determined at 1 bar for fayalite at 1490 K [30, 89.3±1.1 kJ/mol] and that estimated for forsterite at 2163 K is 114±20 kJ/mol [31]. Based on these data, three simplifications are made to estimate the ΔH_{fus} at 20 GPa for the complex Martian mantle mineralogies. The first is that ΔH_{fus} is taken as 110 kJ/mol, which is the value that reproduces the binary forsterite-fayalite liquidus temperature at 1 bar using equation 1 (Figure S14).

This value is intermediate between experimentally determined ΔH_{fus} for both endmembers. The second is that we assume ΔH_{fus} does not change significantly as a function of pressure. The suitability of this assumption may be assessed by examining the $\mathrm{K_D^{Fe-Mg}}$ between olivine and melt as a function of pressure. Experimental data [32, 33] indicate only a marginal effect of pressure on the value of $\mathrm{K_D^{Fe-Mg}}$, which is counteracted by that of temperature. For our

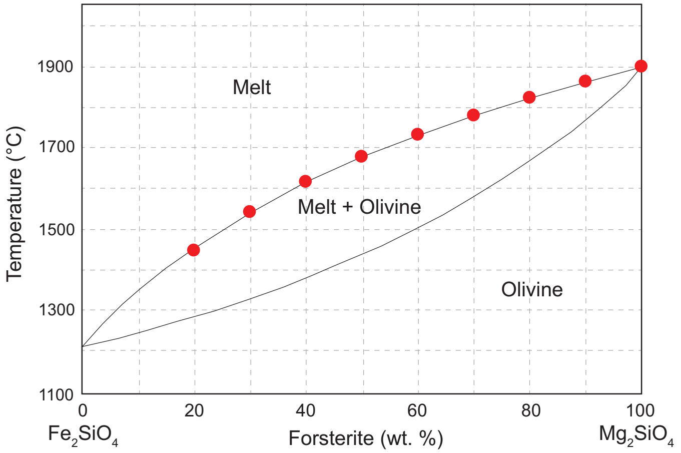

Fig. S14 The forsterite-fayalite binary loop at 1 bar pressure. The thermodynamic properties determined are shown in black, and the approximation of the liquidus curve using eq. 1 for $T_0 = 2163$ K and $\Delta H_{fus} = 110$ kJ/mol shown at 0.1 Mg# intervals are shown in red.

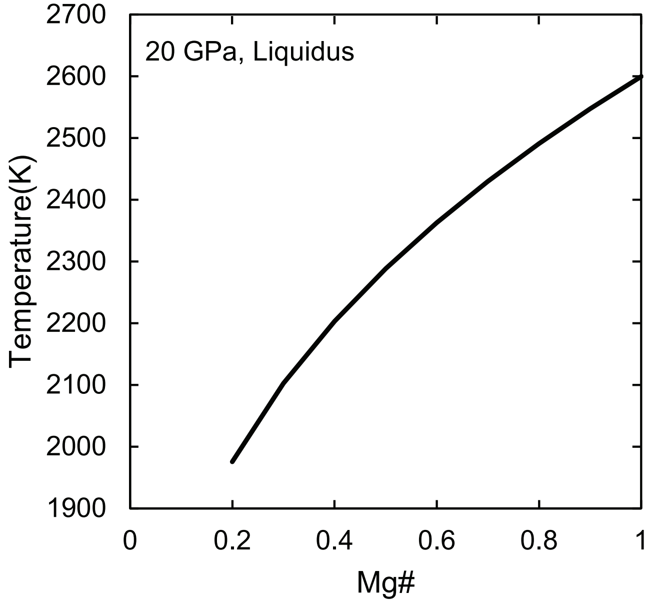

Fig. S15 The estimated liquidus of the Martian mantle with a peridotitic composition at 20 GPa as a function of its Mg# using the cryoscopic equation (eq. 1) at constant $T_0 =$ 2600 K and $\Delta H_{fus} = 110$ kJ/mol.

purposes, the assumption of constant ΔH_{fus}, both as a function of composition and of pressure, is sufficient. The third assumption is that the heat of fusion of olivine is representative of that at the liquidus at the temperature and pressure of interest, which is substantiated by experiments that indicate olivine is a liquidus phase up to ~15 GPa [34, 35].

In order to estimate T_0, the liquidus temperature determined by [34] for a nominally anhydrous peridotite composition in the simplified CaO-MgO-Al_2O_3-SiO_2 (CMAS) system is used. These authors report a liquidus temperature around 2598 K at 20 GPa, for $x_0 = 1$ (i.e., iron-free). A T_0 of 2600 K is therefore taken. The resultant liquidus depression as a function of Mg# is shown in Figure S15. At a nominal pressure of 20 GPa, it shows a ~300 K drop in T from Mg# = 1 to Mg# = 0.5 (2288 K), with a further 300 K drop to Mg# = 0.2. The cryoscopic estimate is consistent with the liquidus temperatures of the Mars-like compositions investigated by [35], who report a liquidus temperature around 2473±100 K at 23 GPa.

References

[1] Durán, C., et al.: Observation of a core-diffracted P-wave from a farside impact with implications for the lower-mantle structure of Mars. Geophysical Research Letters **49**(21), 2022–100887 (2022). https://doi.org/

10.1029/2022GL100887

[2] Khan, A., et al.: Upper mantle structure of Mars from InSight seismic data. Science **373**(6553), 434–438 (2021). https://doi.org/10.1126/science.abf2966

[3] Durán, C., et al.: Seismology on Mars: An analysis of direct, reflected, and converted seismic body waves with implications for interior structure. Physics of the Earth and Planetary Interiors **325**, 106851 (2022). https://doi.org/10.1016/j.pepi.2022.106851

[4] InSight Marsquake Service: Mars Seismic Catalogue, InSight Mission; V11 2022-07-01. ETHZ, IPGP, JPL, ICL, Univ. Bristol (2022). https://doi.org/10.12686/a17

[5] Posiolova, L.V., et al.: Largest recent impact craters on Mars: Orbital imaging and surface seismic co-investigation. Science **378**(6618), 412–417 (2022). https://doi.org/10.1126/science.abq7704

[6] Montalbetti, J.F., Kanasewich, E.R.: Enhancement of Teleseismic Body Phases with a Polarization Filter. Geophysical Journal International **21**(2), 119–129 (1970). https://doi.org/10.1111/j.1365-246X.1970.tb01771.x

[7] Ceylan, S., et al.: Companion guide to the marsquake catalog from InSight, Sols 0–478: Data content and non-seismic events. Physics of the Earth and Planetary Interiors **310**, 106597 (2021). https://doi.org/10.1016/j.pepi.2020.106597

[8] Scholz, J.-R., et al.: Detection, analysis and removal of glitches from insight's seismic data from mars. Earth and Space Science Open Archive, 30 (2020). https://doi.org/10.1002/essoar.10503314.2

[9] Horleston, A.C., et al.: The Far Side of Mars: Two Distant Marsquakes Detected by InSight. The Seismic Record **2**(2), 88–99 (2022). https://doi.org/10.1785/0320220007

[10] Irving, J.C.E., et al.: First observations of core-transiting seismic phases on mars. Proceedings of the National Academy of Sciences **120**(18), 2217090120 (2023). https://doi.org/10.1073/pnas.2217090120

[11] Stähler, S.C., et al.: Seismic detection of the martian core. Science **373**(6553), 443–448 (2021). https://doi.org/10.1126/science.abi7730

[12] Khan, A., Sossi, P.A., Liebske, C., Rivoldini, A., Giardini, D.: Geophysical and cosmochemical evidence for a volatile-rich mars. Earth and Planetary Science Letters **578**, 117330 (2022). https://doi.org/10.1016/j.epsl.2021.

117330

[13] Taylor, G.J.: The bulk composition of Mars. Chemie der Erde / Geochemistry **73**, 401–420 (2013). https://doi.org/10.1016/j.chemer.2013.09.006

[14] Nissen-Meyer, T., et al.: AxiSEM: broadband 3-D seismic wavefields in axisymmetric media. Solid Earth **5**(1), 425–445 (2014). https://doi.org/10.5194/se-5-425-2014

[15] Lognonné, P., et al.: Constraints on the shallow elastic and anelastic structure of Mars from InSight seismic data. Nat. Geosci. **13**, 213–220 (2020). https://doi.org/10.1038/s41561-020-0536-y

[16] Giardini, D., et al.: The Seismicity of Mars. Nat. Geosci. **13**, 205–212 (2020). https://doi.org/10.1038/s41561-020-0539-8

[17] Kim, D., et al.: Improving Constraints on Planetary Interiors With PPs Receiver Functions. Journal of Geophysical Research: Planets **126**(11), 2021–006983 (2021). https://doi.org/10.1029/2021JE006983

[18] Zenhäusern, G., et al.: Low-Frequency Marsquakes and Where to Find Them: Back Azimuth Determination Using a Polarization Analysis Approach. Bulletin of the Seismological Society of America **112**(4), 1787–1805 (2022). https://doi.org/10.1785/0120220019

[19] Tsuno, K., Grewal, D.S., Dasgupta, R.: Core-mantle fractionation of carbon in earth and mars: The effects of sulfur. Geochimica et Cosmochimica Acta **238**, 477–495 (2018). https://doi.org/10.1016/j.gca.2018.07.010

[20] Wasson, J.T., Kallemeyn, G.W., Runcorn, S.K., Turner, G., Woolfson, M.M.: Compositions of chondrites. Philosophical Transactions of the Royal Society of London. Series A, Mathematical and Physical Sciences **325**(1587), 535–544 (1988). https://doi.org/10.1098/rsta.1988.0066

[21] Warren, P.H.: Stable-isotopic anomalies and the accretionary assemblage of the Earth and Mars: A subordinate role for carbonaceous chondrites. Earth and Planetary Science Letters **311**(1), 93–100 (2011). https://doi.org/10.1016/j.epsl.2011.08.047

[22] Liebske, C., Khan, A.: On the principal building blocks of mars and earth. Icarus **322**, 121–134 (2019). https://doi.org/10.1016/j.icarus.2019.01.014

[23] Schiller, M., Bizzarro, M., Fernandes, V.A.: Isotopic evolution of the protoplanetary disk and the building blocks of Earth and the Moon. Nature **555**(7697), 501–510 (2018). https://doi.org/10.1038/nature25990

[24] Lodders, K.: Solar System Abundances and Condensation Temperatures of the Elements. The Astrophysical Journal **591**(2), 1220 (2003). https://doi.org/10.1086/375492

[25] Paquet, M., Sossi, P.A., Moynier, F.: Origin and abundances of volatiles on mars from the zinc isotopic composition of martian meteorites. Earth and Planetary Science Letters **611**, 118126 (2023). https://doi.org/10.1016/j.epsl.2023.118126

[26] Tsuno, K., Frost, D.J., Rubie, D.C.: The effects of nickel and sulphur on the core-mantle partitioning of oxygen in earth and mars. Physics of the Earth and Planetary Interiors **185**(1-2), 1–12 (2011). https://doi.org/10.1016/j.pepi.2010.11.009

[27] Gendre, H., Badro, J., Wehr, N., Borensztajn, S.: Martian core composition from experimental high-pressure metal-silicate phase equilibria. Geochemical Perspectives Letters **21**, 42–46 (2022). https://doi.org/10.7185/geochemlet.2216

[28] Barron, L.M.: Thermodynamic multicomponent silicate equilibrium phase calculations. American Mineralogist: Journal of Earth and Planetary Materials **57**(5-6), 809–823 (1972)

[29] Bertka, C.M., Fei, Y.: Mineralogy of the Martian interior up to core-mantle boundary pressures. Journal of Geophysical Research: Solid Earth **102**(B3), 5251–5264 (1997). https://doi.org/10.1029/96JB03270

[30] Stebbins, J.F., Carmichael, I.S.: The heat of fusion of fayalite. American Mineralogist **69**(3-4), 292–297 (1984)

[31] Navrotsky, A., Ziegler, D., Oestrike, R., Maniar, P.: Calorimetry of silicate melts at 1773 k: measurement of enthalpies of fusion and of mixing in the systems diopside-anorthite-albite and anorthite-forsterite. Contributions to Mineralogy and Petrology **101**(1), 122–130 (1989). https://doi.org/10.1007/BF00387206

[32] Agee, C.B.: Crystal-liquid density inversions in terrestrial and lunar magmas. Physics of the Earth and Planetary Interiors **107**(1), 63–74 (1998). https://doi.org/10.1016/S0031-9201(97)00124-6

[33] Toplis, M.J.: The thermodynamics of iron and magnesium partitioning between olivine and liquid: criteria for assessing and predicting equilibrium in natural and experimental systems. Contributions to Mineralogy and Petrology **149**(1), 22–39 (2005). https://doi.org/10.1007/s00410-004-0629-4

[34] Litasov, K., Ohtani, E.: Phase relations and melt compositions in

CMAS-pyrolite-H_2O system up to 25 GPa. Physics of the Earth and Planetary Interiors **134**(1-2), 105–127 (2002). https://doi.org/10.1016/S0031-9201(02)00152-8

[35] Duncan, M.S., Schmerr, N.C., Bertka, C.M., Fei, Y.: Extending the solidus for a model iron-rich martian mantle composition to 25 gpa. Geophysical Research Letters **45**(19), 10211–10220 (2018). https://doi.org/10.1029/2018GL078182
